# SHORTER trial: protocol for a pragmatic, multicentre, randomised controlled trial of short-duration antibiotic therapy for critically ill patients with sepsis

**DOI:** 10.1136/bmjopen-2026-117142

**Published:** 2026-03-26

**Authors:** Phil Mawson, Miranda Morton, Zoe Walmsley, Rebecca Wafer, Helen C Hancock, Helen Mossop, Sarah Al-Ashmori, Lydia M Emerson, Jamie Smith, Gosha Colquhoun, Hangjian Wu, Cristina Fernandez-Garcia, Laura Ternent, Rashmi Kumar, Margaret Ogden, Laura Loraine, Leia Taylor, Sipho Ndlovu, Phil Hall, Anthony C Gordon, Anthony Rostron, Ronan McMullan, Tim Felton, Timothy Walsh, Daniel Francis McAuley, Paul Dark, A John Simpson, Thomas P Hellyer

**Affiliations:** 1 Newcastle University Translational and Clinical Research Institute, Newcastle upon Tyne, UK; 2 Newcastle Clinical Trials Unit, Population Health Sciences Institute, Newcastle University, Newcastle upon Tyne, UK; 3 Biostatistics Research Group, Population Health Sciences Institute, Newcastle University, Newcastle upon Tyne, UK; 4 The Medical School, University of Exeter, University of Exeter, Exeter, UK; 5 Queen’s University Belfast Wellcome-Wolfson Institute for Experimental Medicine, Belfast, UK; 6 School of Health & Social Care, Edinburgh Napier University, Edinburgh, UK; 7 Division of Research Methodology, Wrocław Medical University, Wrocław, Poland; 8 Health Economics Group, Population Health Sciences Institute, Newcastle University, Newcastle upon Tyne, UK; 9 Patient and Public Involvement Partner, United Kingdom, UK; 10 Newcastle Joint Research Office, Newcastle Upon Tyne Hospitals NHS Foundation Trust, Newcastle upon Tyne, UK; 11 Division of Anaesthetics, Pain Medicine and Intensive Care, Imperial College London, London, UK; 12 Department of Medical Microbiology, Queen’s University Belfast Wellcome-Wolfson Institute for Experimental Medicine, Belfast, UK; 13 Division of Immunology, Immunity to Infection and Respiratory Medicine, University of Manchester, Manchester, UK; 14 Usher Institute of Population Health Sciences, The University of Edinburgh, Edinburgh, UK; 15 Respiratory Medicine Unit, Newcastle Upon Tyne Hospitals NHS Foundation Trust, Newcastle upon Tyne, UK; 16 Department Critical Care, Royal Victoria Infirmary, Newcastle Upon Tyne Hospitals NHS Trust, Newcastle upon Tyne, UK

**Keywords:** INTENSIVE & CRITICAL CARE, Sepsis, Intensive Care Units, Adult intensive & critical care, Antibiotics

## Abstract

**Introduction:**

Sepsis is a life-threatening syndrome resulting from a dysregulated immune response to an infection. Patients with sepsis can become critically ill and require advanced organ support in a hospital critical care setting. Antibiotics are lifesaving in sepsis, but overuse is associated with harm and promotes antimicrobial resistance, a rising global challenge making antibiotic treatment less effective. The prevalence of antibiotic use is very high in patients admitted to critical care. Research indicates that shorter courses of antibiotics are as effective as longer durations in the treatment of certain infections, but uncertainty remains for patients with sepsis. The aim of SHORTER is to investigate whether treating critically ill patients with suspected or confirmed sepsis with a fixed 5-day, short course of antibiotics is clinically and cost-effective compared with standard of care.

**Methods and analysis:**

SHORTER is a pragmatic, multicentre, randomised controlled trial. 2244 adults treated with antibiotics for suspected or confirmed sepsis in a critical care setting will be recruited from 50 UK National Health Service hospitals. Participants will be randomised to either a fixed 5-day index course of antibiotics (intervention) or standard of care duration antibiotics (control). The coprimary outcomes are 28-day mortality (non-inferiority) and antibiotic treatment days (superiority). Secondary outcomes will assess the effect of short-duration antibiotic therapy on 90-day mortality, hospital readmissions, further infection rates and health economic impacts. A process evaluation will be embedded in the trial.

**Ethics and dissemination:**

Favourable ethical opinion has been received from the Wales Research Ethics Committee 4 (Ref: 23/WA/0197) and Scotland A REC (Ref: 24/SS/0013). Results will be publicly disseminated via Patient Public Involvement and Engagement representatives, charities and media, and to the clinical community via professional societies, peer-reviewed publications and conference presentations.

**Trial registration number:**

ISRCTN40090372.

**STRENGTHS AND LIMITATIONS OF THIS STUDY**

- This large-scale randomised controlled trial has been designed to provide a definitive evidence base for the effectiveness of a 5-day antibiotic course for the treatment of sepsis.
- The process evaluation (PE) will provide empirical evidence on the implementation and delivery of this complex intervention trial, contributing to the currently limited PE evidence in this setting.
- A multicentre study across the UK will strengthen generalisability of results.
- Open-labelled trial at risk of performance bias.
- Non-adherence to intervention would bias towards non-inferiority.

## Introduction

### Background

 Sepsis, a syndrome of life-threatening organ dysfunction secondary to infection, is a leading cause of death worldwide.^1^ In England, sepsis accounts for one-third of general adult critical care admissions, equating to approximately 40,000 patients per year, with an estimated cost to the National Health Service (NHS) of £15 billion annually.^2 3^

Treatment of sepsis often requires advanced organ support in a critical care setting and antibiotics which can be lifesaving. Current international and national guidelines recommend initiation of antibiotics within 1 hour of suspected septic shock.^4 5^ While essential in sepsis management, there are risks associated with the potential overuse of antibiotics including adverse side effects experienced by patients and antimicrobial resistance (AMR). The harms associated are concentrated in critical care where antibiotic use far exceeds other hospital settings.^6^ Overuse of antibiotics is associated with deterioration in renal function from drug toxicity,^7^ antibiotic-associated infections such as *Clostridium difficile*,^8^ prolonged intensive care stay and organ support,^9^ and increased mortality.^7^

AMR continues to rise globally. In England, an estimated 65,000 antibiotic-resistant infections were reported in 2019,^10^ and 2024 data show increasing rates with approximately 400 new episodes of antibiotic-resistant bacteraemia weekly.^11^ In Scotland, numbers of antibiotic-resistant bacteraemia have been increasing annually since 2021.^12^ Critically ill patients are vulnerable to healthcare-associated infections, further increasing the risk of infections from resistant pathogens^13^ and the spread of antibiotic-resistant infection to other areas of the hospital.^14 15^

Previous guidelines recommend treatment of sepsis with 7–10 days of antibiotics.^6^ Updated guidelines encourage shorter courses, although such recommendations are regarded as ‘weak’, based on low-quality evidence.^4^ The UK Health Security Agency and the National Institute for Health and Care Excellence promote antibiotic stewardship, encouraging the shortest duration of antibiotics needed and discontinuation of antibiotics if there is no evidence of infection. However, these recommendations overlook individual-level and system-level decision-making factors.^16^ Interventions to optimise antibiotic duration for patients with sepsis are largely biomarker guided, predominantly using procalcitonin but also C-reactive protein (CRP), reducing antibiotic duration during the initial treatment of sepsis by 1–3 days.^17^,^20^ Compliance with these interventions has been low^17^ and clinicians are reluctant to discontinue antibiotics despite algorithm-recommended stopping.^21 22^

Antibiotics are frequently prescribed in fixed course lengths but a review of the evidence in sepsis found no prospective randomised clinical trials of fixed antibiotic duration in critically ill patients with sepsis.^23^ Determining the shortest duration that is safe and effective would represent a widely applicable approach that could significantly reduce antibiotic exposure in patients treated for sepsis. Such are the challenges of delivering complex interventions in the high acuity and risk-averse setting of critical care, a process evaluation (PE) will be essential.^24^

Short-duration antibiotic courses have been trialled in some specific infections including community-acquired pneumonia (CAP),^25^ intra-abdominal infections,^26^ urinary tract infections^27^ and ventilator-associated pneumonia.^28^ Although these trials were not specific to sepsis, outcomes supported shorter antibiotic durations and subsequent analyses indicate that shorter courses of antibiotics may have non-inferior outcomes in patients with sepsis.^27 29^

Overall, current evidence provides proof of concept that short-duration antibiotics may be applied safely in critically ill patients with sepsis. Advances in sepsis pathobiology show that the organ dysfunction is a consequence of a dysregulated immune response^30^ rather than indicating a failure of antibiotics or need for prolonged courses. The SHORTER trial aims to address whether fixed, short-course antibiotics of 5 days are clinically and cost-effective in critically ill patients with sepsis.

## Methods and analysis

### Trial design and setting

SHORTER is a pragmatic, open-label, multicentre, parallel-arm, randomised controlled trial, with an embedded PE to assess the clinical and cost-effectiveness of short, fixed-course antibiotics compared with standard of care in adult patients admitted to critical care with confirmed or suspected sepsis. The trial will be conducted in 50 UK NHS hospitals with critical care units, including both high-dependency units (HDU) and intensive care units (ICU). Patients will be randomised 1:1 to either a 5-day fixed course of antibiotics (intervention) or standard of care duration antibiotics (control) and followed up for 90 days.

### Eligibility criteria

Patients will be considered eligible if they fulfil all the inclusion criteria and none of the exclusion criteria.

#### Inclusion criteria

- Adult patients, aged ≥18 years, treated within a critical care setting (ICU or HDU) for suspected or confirmed sepsis due to either community-acquired or hospital-acquired infections.
- Evidence of new or worsening acute organ dysfunction resulting from suspected or confirmed infection (eg, the treatment or monitoring of an organ dysfunction).
- Antibiotics initiated for suspected or confirmed sepsis, patient is still receiving antibiotics at randomisation and is able to be randomised within 4 days of the initiation of this course of antibiotics.

#### Exclusion criteria

- Comorbidity with immunosuppression (eg, chemotherapy, post-transplantation, maintenance corticosteroids equivalent to >10 mg/day of prednisolone).
- Blood neutrophil count less than 0.5×10^9^ /L secondary to a pre-existing comorbidity.
- Infection source where usual practice involves more than 14 days of antibiotics (eg, undrainable abscess, endocarditis, *Staphylococcus aureus* bacteraemia, osteomyelitis).
- Receiving end-of-life care.
- Life-sustaining treatment expected to be withdrawn within the next 24 hours.
- The clinician responsible for the patient’s care is unable to adhere to the intervention.
- Concomitant enrolment in another interventional trial, except where a coenrolment agreement with SHORTER exists.

### Outcomes

Coprimary outcome measures:

- 28-day all-cause mortality (non-inferiority safety outcome).
- Total antibiotic treatment days measured at 28 days (superiority clinical effectiveness outcome).

Secondary outcome measures:

- 90-day all-cause mortality.
- Suspected clinically relevant antibiotic-associated adverse events during index hospital admission occurring from randomisation up to hospital discharge (or day 90 if the participant has not been discharged at this point).
- Length of critical care unit stay up to 90 days.
- Length of hospital stay up to 90 days.
- Number of further/recurrent infections requiring additional antibiotic courses following index sepsis episode up to 28 days.
- Readmission to critical care or hospital during the 90-day follow-up period.

Secondary outcome measures (health economic):

- Incremental cost per death avoided.
- Incremental cost per quality-adjusted life year (QALY) gained at 90 days.
- Cost-effectiveness acceptability curves (CEACs) to assess the probability of the intervention being considered cost-effective at different willingness-to-pay (WTP) thresholds for a gained QALY at 90 days.
- Average healthcare costs per participant over 90 days following the initiation of antibiotic treatment for each area of resource use.
- Utility scores derived from responses to the EuroQol 5-Dimension 5-Level (EQ-5D-5L) questionnaire at 90 days following the initiation of antibiotic treatment.
- Average QALYs per participant at 90 days following the initiation of antibiotic treatment.
- Incremental cost per QALY gained over the patient’s lifetime.
- CEACs to assess the probability of the intervention being considered cost-effective at different WTP thresholds for a gained QALY over the participants’ lifetime.

Secondary outcome measure (PE)

- To evaluate the implementation and delivery of both the trial and the intervention, and to understand how contextual factors impact upon successful delivery.

### Participant identification

All adult patients (≥18 years) admitted to receive critical care will be screened. Patients will be identified as potentially eligible if they are being treated with antibiotics and between treatment days 1 and 4 of the index antibiotic course for either suspected or confirmed sepsis.

### Consent

Consent will be sought prior to any trial-specific activity. Critically ill patients may lack the capacity to provide informed consent, and this will be assessed and documented prior to consent. The consent processes, accounting for capacity and location of participating centre, are summarised in table 1 (see onlinesupplemental files 1^5^ consent forms).

**Table 1:** Summary of approaches to consent

Patients with capacity	Patient approached directly and provided with Patient Information Sheet (PIS). Patient provides informed consent by signing the Informed Consent Form.
Patients lacking capacity (England, Wales and Northern Ireland)	Assent sought as per the Mental Capacity Act 2005 (England and Wales) and the Mental Capacity Act 2016 (Northern Ireland). Personal Consultee identified; this is an individual who either cares for the patient or who has an interest in the patient’s welfare but is not doing so for renumeration or acting in a professional capacity. Often this will be the next of kin. If there is no suitable personal consultee, a medical professional who is independent of the research team can act as a Nominated Consultee. Consultee provided with Consultee Information Sheet. Consultee provides assent by signing a Consultee Declaration Form. Consultees informed that the participant’s consent will be sought if capacity is regained.
Patients with incapacity (Scotland)	Consent sought as per the Adults with Incapacity Act 2000 (Scotland). Legal Representative identified; this may be a Welfare Attorney, Welfare Guardian or nearest relative. No professional Legal Representative consent is permitted. Legal Representative provided with Legal Representative Information Sheet. Legal Representative provides consent by signing the Legal Representative Consent Form. Legal Representatives informed that the participant’s consent will be sought if capacity is regained.
Participants who lose capacity	Ongoing consent will not be assumed; at consent, patients are asked to identify a nominated representative who is able to provide ongoing consent monitoring in the event of loss of capacity. If capacity is lost, ongoing participation in the trial will be discussed with the nominated representative.
Participants who regain capacity	If capacity is regained during trial participation, the participant will be informed of their involvement in the trial and provided with a Recovered Capacity PIS. If the participant agrees to ongoing participation, they will be asked to provide informed consent retrospectively. If the participant does not agree to ongoing participation, they will be withdrawn from the trial.

Participants, or their representative, have the right to withdraw from all or some aspects of the trial at any time.

### Randomisation

Patients will be randomised 1:1 to either the intervention or control using the trial database Sealed Envelope Red Pill System (London, UK). This is a central, secure, 24-hour, web-based randomisation system with concealed allocation.

The allocation sequence will be computer generated using random permuted blocks and stratified by recruiting centre and by community-acquired versus hospital-acquired infection (defined as occurring 48 hours after admission to hospital).

### Blinding

This is an open-label trial with no blinding of clinical teams or outcome assessors. Blinding was judged not to be feasible due to the importance of the clinical teams being aware of antibiotic therapy that patients are receiving. The first version of the Statistical Analysis Plan was written prior to the statistical team accessing unblinded data.

### Intervention

The intervention is a 5-day, fixed, course of antibiotic treatment. The index course is the initial antibiotic course prescribed for the active treatment of suspected or confirmed sepsis at the point of randomisation. For participants randomised to the intervention, a stop date and time will be confirmed.

The intervention only relates to duration. The choice or combination of antibiotics will be decided by clinical teams aligned to local antibiotic guidelines. Sites will not use any biomarker-guided antibiotic duration algorithm to extend the antibiotic duration beyond 5 days in the intervention arm. However, if antibiotics for sepsis are inadequate due to a blood culture-proven resistant organism, then the index antibiotic course start date and time can be reset at the initiation of adequate antibiotics.

All other aspects of usual care such as multidisciplinary antibiotic stewardship will continue in the intervention arm and additional antibiotics may be given after the initial 5-day course to ensure patient safety if clinically indicated.

### Control

The duration of antibiotics will be according to the standard of care. This will be guided by the treating clinician, local antibiotic guidelines and usual antibiotic stewardship practice.

### Assessment of compliance

Compliance with the intervention course length as well as overall trial group separation of number of antibiotic treatment days will be monitored by the Trial Management Group throughout the trial. This approach allows reasons for non-compliance to be explored early, enabling early discussion with sites when issues arise which are not compliant with the trial protocol.

### Participant follow-up and data collection

Trial day 1 is the start of the index course of antibiotics for suspected/confirmed sepsis which will occur prerandomisation. Participants will be followed from trial day 1 up to day 90. Demographic and clinical data associated with participants’ hospital admission will be collected during the index antibiotic treatment course and index hospital stay (or day 90 if the participant has not been discharged at this point).

Data relating to 28-day and 90-day all-cause mortality, the number of antibiotic treatment days at 28 days, further occurrence or re-occurrence of infections requiring antibiotic treatment at 28 days, and readmissions up to day 90 will be collected. Health-related quality of life, measured by the EQ-5D-5L questionnaire, will be completed by participants or their proxy at hospital discharge and day 90. A Healthcare Utilisation and Time and Travel Questionnaire will also be completed by participants or their proxy at day 90 (see online supplemental file 6 Schedule of Events).

Where possible, follow-up data may be collected about participants via hospital records, general practitioners or clinical data repositories such as the Intensive Care National Audit & Research Centre (ICNARC).

### Participant safety and reporting

The trial will test the short-course antibiotic intervention because there is increasing evidence of the harms associated with antibiotic overuse. However, there remains a risk of undertreatment, and the short course may not be effective for some patients. The trial is pragmatic and embeds the intervention in usual antibiotic stewardship practice. The clinical team will continue to have oversight and can make decisions necessary to maintain patient safety.

As the patient population is critically ill, it is expected that many participants will experience adverse events (AEs). AEs considered to be associated with antibiotic use will be reported. Other AEs will be excluded from reporting, if they are:

- Part of the expected natural history of the primary disease process.
- Consistent with critical illness.
- Common complications of surgery.
- Associated with the initial suspected/confirmed infection that led to the participant’s inclusion in the trial.
- Already recorded as part of the trial outcomes (ie, organ failure, death or further infection requiring antibiotics).

Serious adverse events (SAEs) that do not meet exclusion criteria will be reported within 24 hours of awareness; this includes any SAE that is judged to have a causal relationship to the trial intervention. As there is no reference safety information of known expected reactions for the intervention, any SAE deemed related to the intervention will be classified as an unexpected related serious event and reported to Research Ethics Committee within regulatory timeframes.

### Data management

Data will be collected via an electronic case report form (eCRF) in a trial-specific, secure, web-based Clinical Data Management System using Sealed Envelope’s Red Pill System (London, UK). Participant data will be recorded using a unique identification number, assigned at screening.

The chief investigator has overall responsibility for data collection, quality and retention and will be the custodian of the final dataset. Data will be handled, computerised and stored in accordance with the Data Protection Act 2018, the latest Directive on Good Clinical Practice (2005/28/EC) and local hospital policy. Data will be managed and validated as per trial-specific Data Management and Data Validation Plans. Data integrity and trial conduct will be monitored in accordance with the trial Monitoring Plan.

Data will be shared with ICNARC, or other local equivalents, for the purpose of data linkage to collect critical care outcome measures. Data linkage will be performed using NHS/Community Health Index/Health & Safety/local equivalent number, date of birth and sex at birth or audit data-specific identifiers (eg, Case Mixed Programme number for ICNARC requests) and date of randomisation.

### Sample size

The target sample size is 2244 participants, accounting for a dropout rate of 5%. The sample size is based on assessing non-inferiority for 28-day all-cause mortality, assuming a mortality rate of 24%,^31^ with 90% power, a one-sided type 1 error rate of 2.5% and a non-inferiority margin of 6%. The sample size will also have 90% power to detect a 1.1-day difference in total antibiotic treatment days to 28 days, assuming a SD of 7.7^19^ and a two-sided type 1 error rate of 5%.

### Analysis

#### Statistical methods

For the analysis of 28-day all-cause mortality, a complete case analysis will be performed in the intention-to-treat (ITT) population. Data for participants who withdraw consent for continued collection of routinely available data prior to day 28 will not be included in the analysis. To allow for centre effects, the absolute risk difference (intervention − control) and 95% CI will be obtained from a binomial generalised estimating equation model with identity link function and exchangeable correlation structure, adjusted for other stratification factors, following recommended methods.^32^ Alternative methods will be employed should the model fail to converge. Non-inferiority will be demonstrated if the upper limit of the 95% CI is below the non-inferiority margin of 6%. A one-sided p value for non-inferiority will be calculated using a Wald test with a critical threshold of 0.025.

A supplementary analysis will estimate the relative difference between randomised treatment groups using a mixed-effects logistic regression model with centre included as a random effect and other stratification factors as fixed effects.

Views on an acceptable non-inferiority margin for the primary safety outcome may vary by stakeholder, or if the observed event rate differs from that assumed. To address this, we will provide non-inferiority acceptability curves^33^ showing the probability that short-duration treatment is non-inferior to standard of care for a range of non-inferiority thresholds.

If non-inferiority is demonstrated for 28-day mortality, we will analyse for a between-arm reduction in total antibiotic treatment days to 28 days using a mixed-effects linear regression model, with centre included as a random effect and other stratification factors as fixed effects. This analysis will be conducted in the ITT population. A supplementary analysis will also be performed to estimate the survivor average causal effect; the treatment effect in the subpopulation of participants who would have survived to day 28 regardless of treatment allocation.

Subgroup analyses of the coprimary outcomes will be carried out on the following subgroups:

- Culture-negative sepsis, defined as no positive culture results from samples taken in relation to the index course of antibiotics for sepsis.
- Positive blood culture, defined as a positive blood culture from a sample taken in relation to the index course of antibiotics for sepsis.
- Site of infection including CAP, urinary tract infection and intra-abdominal infection.
- Septic shock status.^1^
- Baseline severity of illness.

Secondary outcomes will be analysed similarly to the coprimary outcome, using mixed-effects regression models suitable to the type of outcome, and adjusted for stratification factors. 90-day all-cause mortality will be analysed in the same way as for the 28-day mortality outcome. Length of critical care unit and hospital stay (time to hospital discharge) will be compared by arm using time-to-event models. Further infection and readmission to critical care will be analysed using logistic regression and time-to-event models.

Full details of all planned analyses are provided in online supplemental file 7 SHORTER Statistical Analysis Plan.

#### Health economics

The economic analysis will include a within-trial economic evaluation to assess the incremental cost per death avoided, and per QALY gained, at 90 days following the initiation of antibiotic treatment. If appropriate, a longer-term economic model will extrapolate costs and outcomes beyond the 90-day follow-up period. The perspective of the evaluation is that of the UK NHS and personal and social services, and wider societal costs. All analyses will be conducted following best practice guidance.^34 35^

The within-trial analysis will include data on resource use collected for every trial participant. Intervention costs will include details of the antibiotic treatment and costs associated with the participants’ hospital stay during their treatment as recorded on the eCRF. Healthcare utilisation data will be collected via a Health Care Utilisation Questionnaire (HCUQ), administered at 90 days following the initiation of antibiotic treatment. Participant-related costs will also be collected in the Time and Travel Questionnaire administered at day 90. Unit costs associated with antibiotics will be identified from the British National Formulary. Hospital stay unit costs will be sourced from the NHS National Cost Collection Database. Unit costs associated with follow-up healthcare resource use will be collected from routine sources.^36 37^ Intervention and healthcare resource use data will be combined with the relevant unit costs to produce a cost for each trial participant.

The within-trial analysis will compare changes in mortality and in QALYs at day 90. First, the number of deaths measured at day 90 will be used as a measure of effectiveness for the cost-effectiveness analysis. Second, QALYs derived from the EQ-5D-5L questionnaire^38^ at day 90 will be used as the second effectiveness measure for the cost-utility analysis.

Results will be presented as point estimates of mean incremental cost per death avoided and mean incremental cost per QALY gained at 90 days, and as cost-effectiveness acceptability curves. Deterministic sensitivity analysis will be used to address any uncertainty in the assumptions used in the base-case analysis. A stochastic sensitivity analysis, using the bootstrapping technique,^39^ will explore the impact of the statistical imprecision surrounding estimates of costs, effects and cost-effectiveness. The bootstrapped results will be graphically represented using the cost-effectiveness and CEACs.^35 40^ The cost-effectiveness plane is used to illustrate the distribution of incremental costs and incremental effects from which we can identify the uncertainty in our results. CEACs will allow us to identify the management strategy that maximises net benefits at each WTP value for an additional unit of health effect (ie, death avoided and QALYs gained).

#### Process evaluation

A mixed-methods PE will be conducted alongside the trial to examine processes relating to the trial and intervention, including their implementation and to delivery, and how these processes operate within the trial context. The PE will be informed by the trial logic model underpinning the intervention and will be designed to test key theoretical assumptions relating to how the intervention is expected to produce outcomes under trial conditions.

The PE will be conducted across three phases of the trial, capturing pretrial practice, during-trial delivery and end-of-trial delivery.^24^ It will examine whether hypothesised mechanisms are delivered as intended and how contextual-level, organisational-level and participant-level factors influence observed processes and outcomes. Data collection will address core PE domains including context, fidelity, dose and reach.

The Conceptual Framework for Implementation Fidelity and Normalisation Process Theory will be used as the analytical lens, guiding analysis of qualitative and quantitative data. Analysis will be structured to support interpretation of trial outcomes, explain variation in effects and inform future implementation and scale-up beyond the trial context where appropriate. A detailed PE protocol describing these methods is currently under submission for publication.^41^

### Patient and public involvement

Patient and public involvement (PPI) has been integral to the development of the trial from its inception. The importance of the research question is recognised by two PPI groups; Voice and ICU-steps.^42 43^ PPI coapplicants with lived experience of antibiotic use and the need for antibiotic stewardship have contributed to the development and amendments of the protocol and patient-facing documentation. PPI coapplicants are active members of the Trial Management Group, and an independent PPI partner is a member of the Trial Steering Committee.

### Trial status

At the time of writing, the trial has been open to recruitment since January 2024. The status of the trial can be viewed via ISRCTN. This manuscript is based on protocol V.6.0 dated 24 September 2025.

### Trial oversight

The Newcastle upon Tyne Hospitals NHS Foundation Trust is the sponsor. The Trial Steering Committee is comprised of independent members and is responsible for the review of aggregate safety data collected to date to identify any trends and liaise with the Data Monitoring and Ethics Committee (DMEC) regarding safety issues. The DMEC is comprised of independent members and is responsible for the review of safety data by randomised treatment group to determine patterns and trends of events, or to identify safety issues which would not be apparent on a case-by-case basis.

## Ethics and dissemination

Favourable opinions from the Wales Research Ethics Committee 4 (Ref: 23/WA/0197) and Scotland A Research Ethics Committee (Ref: 24/SS/0013) aligned to the Adults with Incapacity (Scotland) Act 2000 are in place. The trial has Health Research Authority approval (IRAS: 317788) to run in the UK and is adopted onto the UK Clinical Research Network (CRN) portfolio (CPMS 317788).

Trial results will be publicised throughout the clinical community, PPI groups and society more widely. Findings will be published in peer-reviewed publications.

## Supplementary material

10.1136/bmjopen-2026-117142 online supplemental file 1

10.1136/bmjopen-2026-117142 online supplemental file 2

10.1136/bmjopen-2026-117142 online supplemental file 3

10.1136/bmjopen-2026-117142 online supplemental file 4

10.1136/bmjopen-2026-117142 online supplemental file 5

10.1136/bmjopen-2026-117142 online supplemental file 6

10.1136/bmjopen-2026-117142 online supplemental file 7
