# Supplementary material for: SHORTER trial: protocol for a pragmatic, multicentre, randomised controlled trial of short-duration antibiotic therapy for critically ill patients with sepsis
Source: BMJ Open. 2026 Mar 26;16(3):e117142. doi: 10.1136/bmjopen-2026-117142 (PMC13034387; doi:10.1136/bmjopen-2026-117142)
Supplement: online supplemental file 1 [file bmjopen-16-3-s001.docx]

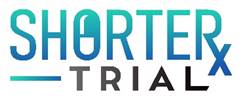


A randomised controlled trial of SHORT duration antibiotic thERapy for critically ill patients with sepsis

**INFORMED CONSENT FORM**

**(Non-Scottish sites)**

**V6, 17 December 2024**

|  |  |  |  |  |  |
| --- | --- | --- | --- | --- | --- |

**Trial ID:**

**Principal Investigator: _______________________________________**

**Please INITIAL boxes if you agree**:

| 1. | I confirm that I have read and understood the patient information sheet dated _ _ / _ _ / _ _ _ _ (version __.__). I have had the opportunity to consider the information, ask questions and have had these answered satisfactorily. |  |
| --- | --- | --- |
| 2. | I understand that my participation is voluntary and that I am free to withdraw at any time without giving any reason and without my medical care or legal rights being affected. I understand that all data already collected about me up to the point of withdrawal will be retained. |  |
| 3. | I understand that relevant information taken from my medical records and routine data collection during the trial will leave my local NHS Trust. This includes my date of birth, sex at birth, postcode, employment status and ethnicity which will be stored in the central trial database managed by Newcastle University. I understand that my data will be stored securely and managed confidentially as part of this trial. |  |
| 4. | I understand that my medical records and data may be looked at by responsible individuals from Newcastle University, regulatory authorities or the NHS Trust. My personal identity and private information will be anonymised. I give permission for these individuals to have access to my records and data. |  |
| 5. | I understand that any personal information collected about me for the trial will be kept confidential and not be made public. I understand that data from the trial will be published in medical journals, at research meetings and shared with other researchers, including researchers potentially outside the United Kingdom (UK) in the European Economic Area (EEA). I understand that data from the trial will be de-identified and that I will not be directly identified in the published results. |  |
| 6. | I agree to the research team requesting information about my health and hospital admissions (up to the 90 day follow up) from routine sources including Intensive Care National Audit & Research Centre (ICNARC) or other local equivalents. |  |
| 7. | I understand that personally identifiable data including my NHS/H&C number, date of birth and sex at birth will be collected and stored by Newcastle University to link with the information held and maintained by central UK NHS bodies and intensive care audit bodies. I give permission for these individuals to store this information until the end of the trial after which it will be destroyed. |  |
| 8. | I understand that anonymous information collected about me could be used to support other research in the future and may be shared anonymously with other research projects and researchers, without my personal identity and contact details. |  |
| 9. | I understand that the information provided in this trial is being managed by the Newcastle Clinical Trials Unit, which is part of Newcastle University. |  |
| 10. | I understand that data collected about me will have identifying details removed and will be archived (in accordance with the Data Protection Act) in a secure location for five years after the end of the trial. |  |
| 11. | I agree to my General Practitioner being informed of my involvement in the trial and agree to the exchange of necessary information about me between my GP and the research team. |  |
| 12. | I agree to take part in the SHORTER trial as outlined in the Patient Information Sheet, including follow up in approximately 3 months’ time (90 days) to complete trial questionnaires. |  |
| 13. | (Optional) I would like to receive a copy of the final summary of the trial. | Yes  No |

Name of patient*: Date*: Signature*:

_________________________ ______________ _________________________

Name of person taking consent: Date: Signature:

_________________________ ______________ _________________________

****If the participant cannot write on or read this form, please ask their next of kin or a member of staff who is independent of the research team (this may be a nurse or a doctor) to read and complete the following on their behalf:***

I have no involvement in this research trial, and I attest that the information concerning this research was accurately explained to the participant in language they can understand, and that informed consent was given freely by the participant.

Name of witness: Date: Signature:

_________________________ ______________ _________________________

**Nominated contact *(to be completed by the participant or on their behalf by the person taking consent if the participant is unable to write on or read this form)*:**

I understand that I am currently unwell and that my ability to answer questions and discuss this research may change during my treatment. If I am unable to answer for myself, I would like you to discuss my continued involvement in the SHORTER trial with: _________________________________________________________________

Their relationship to me is: _______________________________________________

The best way to contact them is: __________________________________________

**IF** you have chosen to receive a copy of the final summary of the trial, please select the best way to send to this information to you:

By Post

Via Email to: _________________________________________

**(Original to be retained and filed in the Investigator Site File, one copy for the patient, and one copy filed in the patient medical records)**
