# Supplementary material for: SHORTER trial: protocol for a pragmatic, multicentre, randomised controlled trial of short-duration antibiotic therapy for critically ill patients with sepsis
Source: BMJ Open. 2026 Mar 26;16(3):e117142. doi: 10.1136/bmjopen-2026-117142 (PMC13034387; doi:10.1136/bmjopen-2026-117142)
Supplement: online supplemental file 2 [file bmjopen-16-3-s002.docx]

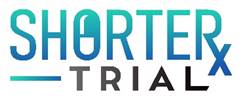


A randomised controlled trial of SHORT duration antibiotic thERapy for critically ill patients with sepsis

**CONSULTEE DECLARATION FORM**

**(Non-Scottish sites)**

**V4.0, 12 March 2024**

|  |  |  |  |  |  |
| --- | --- | --- | --- | --- | --- |

**Trial ID:**

**Principal Investigator: _______________________________________**

**Please INITIAL boxes if you agree**:

| 1. | I confirm that I have been consulted about ___________­­­_____­­­­­­­ participation in this research trial. I confirm that I have read and understood the Consultee Information Sheet dated _ _ / _ _ / _ _ _ _ (version __.__). I have had the opportunity to consider the information, ask questions and have had these answered satisfactorily. |  |
| --- | --- | --- |
| 2. | In my opinion they would have no objection to taking part in the SHORTER trial. |  |
| 3. | I understand that their participation is voluntary and that I can withdraw them at any time without giving any reason and without their medical care or legal rights being affected. I understand that all data already collected about them up to the point of withdrawal will be retained. |  |
| 4. | I understand that relevant information taken from their medical records and routine data collection during the trial will leave their local NHS Trust. This includes their date of birth, sex at birth, postcode, employment status and ethnicity which will be stored in the central trial database managed by Newcastle University. I understand that their data will be stored securely and confidentially as part of the trial. |  |
| 5. | I understand that their medical records and data may be looked at by responsible individuals from Newcastle University, regulatory authorities or the NHS Trust. Their personal identity and private information will be anonymised. I give permission for these individuals to have access to their records and data. |  |
| 6. | I understand that any personal information collected about them for the trial will be kept confidential and not be made public. I understand that data from the trial will be published in medical journals, at research meetings and shared with other researchers, including researchers potentially outside the United Kingdom (UK) in the European Economic Area (EEA). I understand that data from the trial will be de-identified and that they will not be directly identified in the published results. |  |
| 7. | I agree to the research team requesting information about their health and hospital admissions (up to the 90 day follow up) from routine sources including Intensive Care National Audit & Research Centre (ICNARC) or other local equivalents. |  |
| 8. | I understand that personally identifiable data including their NHS/H&C number, date of birth and sex at birth will be collected and stored by Newcastle University to link with the information held and maintained by central UK NHS bodies and intensive care audit bodies. I give permission for these individuals to store this information until the end of the trial when it will be destroyed. |  |
| 9. | I understand that anonymous information collected about them could be used to support other research in the future and may be shared anonymously with other research projects and researchers, without their personal identity and contact details. |  |
| 10. | I understand that the information provided in this trial is being managed by the Newcastle Clinical Trials Unit, which is part of Newcastle University. |  |
| 11. | I understand that data collected about them will have identifying details removed and be archived (in accordance with the Data Protection Act) in a secure location for five years after the end of the trial. |  |
| 12. | I agree to their General Practitioner being informed of their involvement in the trial and agree to the exchange of necessary information about them between their GP and the research team. |  |
| 13. | I agree to the person I am a Consultee for taking part in the SHORTER trial as outlined in the Consultee Information Sheet, including for them to have a follow up in approximately 3 months’ time (90 days) to complete trial questionnaires. |  |

Name of Consultee: Date: Signature:

_________________________ ______________ _________________________

Relationship to participant:

_____________________________________________________________________

Name of person taking consent: Date: Signature:

_________________________ ______________ _________________________

**(Original to be retained and filed in the Investigator Site File, one copy for the Consultee, one copy for the patient (once capacity is recovered) and one copy filed in the patient medical records)**
