# Supplementary material for: SHORTER trial: protocol for a pragmatic, multicentre, randomised controlled trial of short-duration antibiotic therapy for critically ill patients with sepsis
Source: BMJ Open. 2026 Mar 26;16(3):e117142. doi: 10.1136/bmjopen-2026-117142 (PMC13034387; doi:10.1136/bmjopen-2026-117142)
Supplement: online supplemental file 4 [file bmjopen-16-3-s004.docx]

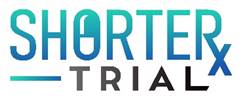


A randomised controlled trial of SHORT duration antibiotic thERapy for critically ill patients with sepsis

**WELFARE ATTORNEY/WELFARE GUARDIAN/NEAREST RELATIVE CONSENT FORM**

**SCOTLAND V2.0, 029 February 2024**

|  |  |  |  |  |  |
| --- | --- | --- | --- | --- | --- |

**Trial ID:**

**Principal Investigator: _______________________________________**

**Please INITIAL boxes if you agree**:

| 1. | I confirm that I have read and understood the Welfare Attorney/Welfare Guardian/Nearest Relative Information Sheet dated _ _ / _ _ / _ _ _ _ (version __.__). I have had the opportunity to consider the information, ask questions and have had these answered satisfactorily. |  |
| --- | --- | --- |
| 2. | In my opinion the person I am consenting for would have no objection to taking part in the SHORTER trial. |  |
| 3. | I understand that the person I am consenting for’s participation is voluntary and that I can withdraw them at any time without giving any reason and without their medical care or legal rights being affected. I understand that all data already collected about them up to the point of withdrawal will be retained. |  |
| 4. | I understand that relevant information taken from the person I am consenting for’s medical records and routine data collection during the trial will leave their local NHS Trust. This includes their date of birth, sex at birth, postcode, employment status and ethnicity which will be stored in the central trial database managed by Newcastle University. I understand that their data will be stored securely and confidentially as part of the trial. |  |
| 5. | I understand that the person I am consenting for’s medical records and data may be looked at by responsible individuals from Newcastle University, regulatory authorities or the NHS Trust. Their personal identity and private information will be anonymised. I give permission for these individuals to have access to their records and data. |  |
| 6. | I understand that any personal information collected for the trial about the person I am consenting for will be kept confidential and not be made public. I understand that data from the trial will be published in medical journals, at research meetings and shared with other researchers, including researchers potentially outside the United Kingdom (UK) in the European Economic Area (EEA). I understand that data from the trial will be de-identified and that they will not be directly identified in the published results. |  |
| 7. | I agree to the research team requesting information about the person I am consenting for’s health and hospital admissions (up to the 90 day follow up) from routine sources including NHS England, Intensive Care National Audit & Research Centre (ICNARC), The Scottish Intensive Care Society Audit Group (SICSAG) or other local equivalents |  |
| 8. | I understand that personally identifiable data including the person I am consenting for’s CHI number, date of birth and sex at birth will be collected and stored by Newcastle Clinical Trials Unit to link with their information held and maintained by NHS England, other central UK NHS bodies and intensive care audit bodies. I give permission for these individuals to store this information until the end of the trial when it will be destroyed |  |
| 9. | I understand that anonymous information collected about the person I am consenting for could be used to support other research in the future and may be shared anonymously with other research projects and researchers, without their personal identity and contact details. |  |
| 10. | I understand that the information provided in this trial is being managed by the Newcastle Clinical Trials Unit, which is part of Newcastle University. |  |
| 11. | I understand that data collected about the person I am consenting for will have identifying details removed and be archived (in accordance with the Data Protection Act) in a secure location for five years after the end of the trial. |  |
| 12. | I agree to the person I am consenting for’s General Practitioner being informed of their involvement in the trial and agree to the exchange of necessary information about them between their GP and the research team. |  |
| 13. | I agree to my relative/person that I am a Welfare Attorney or Welfare Guardian for, taking part in the SHORTER trial as outlined in the Welfare Attorney/Welfare Guardian/Nearest Relative Information Sheet, including for them to have a follow up in approximately 3 months’ time (90 days) to complete trial questionnaires. |  |

I confirm that I am the Welfare Attorney, Welfare Guardian or Nearest Relative for:

_____________________________________________________________________

Relationship to patient:

_____________________________________________________________________

Name of person giving consent: Date: Signature:

_________________________ ______________ _________________________

Name of Person taking consent: Date: Signature:

_________________________ ______________ _________________________

**(Original to be retained and filed in Investigator Site File, one copy for Welfare Attorney/Welfare Guardian/Nearest Relative, one copy for the patient (once capacity is recovered) and one copy filed in patient medical records)**
