# Supplementary material for: SHORTER trial: protocol for a pragmatic, multicentre, randomised controlled trial of short-duration antibiotic therapy for critically ill patients with sepsis
Source: BMJ Open. 2026 Mar 26;16(3):e117142. doi: 10.1136/bmjopen-2026-117142 (PMC13034387; doi:10.1136/bmjopen-2026-117142)
Supplement: online supplemental file 6 [file bmjopen-16-3-s006.docx]

Schedule of Events

| *Antibiotics for sepsis* | *Day 1* | *Day 2* | *Day 3* | *Day 4* | *Day 5* | *Day 6 – Day 14 (according to course length)* | | |  | | |
| --- | --- | --- | --- | --- | --- | --- | --- | --- | --- | --- | --- |
| **Trial day** | **Day 1** | **Day 2** | **Day 3** | **Day 4** | **Day 5** | **Day 6** | **Day 7** | **Day 7-28** | | **Hospital Discharge** | **Day 90** |
| Record date and time of first dose of antibiotics for sepsis | X | | | |  |  |  |  | |  |  |
| Screening & Identification | Screening of patients on antibiotics for sepsis | | | |  |  |  |  | |  |  |
| Consent | Patient consent (or Consultee declaration/Legal Representative consent) | | | |  |  |  |  | |  |  |
| Randomisation | Following consent on days 1-4 | | | |  |  |  |  | |  |  |
| Add antibiotic stop date and time* to prescribing system for participants randomised to short-course antibiotic therapy (intervention) | X (as soon as possible following randomisation) | | | |  |  |  |  | |  |  |
| Baseline data collection | X | | | |  |  |  |  | |  |  |
| Laboratory data collection | X^+/-^ |  | X^+/-^ |  | X^+/-^ |  | X^+/-^ |  | | X^+/-^ |  |
| Sequential Organ Failure Assessment (SOFA) score | X^+/-^ |  |  |  | X^+/-^ |  | X^+/-^ |  | |  |  |
| Functional Comorbidity Index | X |  |  |  |  |  |  |  | |  |  |
| Acute Physiology and Chronic Health Evaluation (APACHE) II Score | Within 24 hours of admission to critical care | | | |  |  |  |  | |  |  |
| Antibiotic stop date for participants randomised to short-course antibiotic therapy (intervention) |  |  |  |  | X*^$^ |  |  |  | |  |  |
| Antibiotic stop date for participants randomised to standard care (control) | Antibiotic duration as per standard of care | | | | | | | | |  |  |
| Assessment of duration of initial antibiotic course |  |  |  |  | Between days 5-14 | | | | |  |  |
| All-cause mortality assessment |  |  |  |  |  |  |  | X | |  | X |
| Antibiotic treatment days to 28 days |  |  |  |  |  |  |  | X | |  |  |
| Adverse Event and Serious Adverse Event reporting | Reported from randomisation to hospital discharge^€,α^ | | | | | | | | | |  |
| Length of critical care unit stay |  |  |  |  |  |  |  |  | | X |  |
| Length of hospital stay |  |  |  |  |  |  |  |  | | X |  |
| Further/re-occurrence of infection requiring antibiotic courses |  |  |  |  |  |  |  | X | |  |  |
| Re-admission to hospital |  |  |  |  |  |  |  |  | |  | X |
| Re-admission to critical care |  |  |  |  |  |  |  |  | |  | X |
| EQ-5D-5L [36] |  |  |  |  |  |  |  |  | | X^†^ | X^¥^ |
| Healthcare utilisation questionnaire ^#^ |  |  |  |  |  |  |  |  | |  | X^¥^ |
| Time and Travel Questionnaire ^#^ |  |  |  |  |  |  |  |  | |  | X^¥^ |

+/- Assessment may take place within +/- 1 day

* As per instruction of antibiotic stop date and time given at randomisation

$Depending on the start time of antibiotics, this may occur on day 6 in practice

€ These data do not need to be collected past day 90, even if the participant remains a hospital in-patient

α Collection of these data does not resume if a participant is readmitted to the same participating site prior to day 90

† Completed by participants or by proxy at hospital discharge (from -2 to + 7 days)

¥ Completed by participants or by proxy at day 90 (+ 14 days)

# Bespoke questionnaires available upon request
