# Supplementary material for: SHORTER trial: protocol for a pragmatic, multicentre, randomised controlled trial of short-duration antibiotic therapy for critically ill patients with sepsis
Source: BMJ Open. 2026 Mar 26;16(3):e117142. doi: 10.1136/bmjopen-2026-117142 (PMC13034387; doi:10.1136/bmjopen-2026-117142)
Supplement: online supplemental file 7 [file bmjopen-16-3-s007.docx]

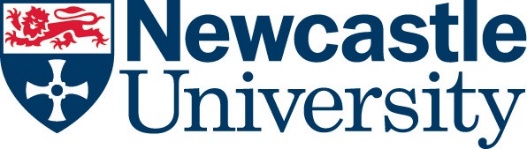
Population Health Sciences Institute

Biostatistics Research Group

A randomised controlled trial of **SHORT** duration antibiotic th**ER**apy for critically ill patients with sepsis (SHORTER)

Statistical Analysis Plan

SAP Version number: 1.0

SAP Date: 22/05/2024

This statistical analysis plan is based on protocol version 4.0 [12/03/2024]

ISRCTN Number: 40090372

IRAS Number: 317788

REC Reference: 23/WA/0197

Sponsor: The Newcastle upon Tyne Hospitals NHS Foundation Trust

Sponsor protocol number: 10238

Funder: NIHR HTA Programme

Funder reference number: NIHR134101

**Abbreviations**

| AEs | Adverse Events |
| --- | --- |
| AIC | Akaike information criterion |
| AS1 | Analysis Set 1 |
| AS2 | Analysis Set 2 |
| AS3 | Analysis Set 3 |
| BIC | Bayesian information criterion |
| CONSORT | Consolidated Standards of Reporting Trials |
| FDA | Food and Drug Administration |
| HR | Hazard ratio |
| HTA | Health technology assessment |
| IDMC | Independent Data Monitoring Committee |
| ITT | Intention to treat |
| IQR | Interquartile range |
| LTFU | Lost to follow up |
| MAR | Missing at Random |
| MI | Multiple Imputation |
| MRSA | Methicillin-resistant Staphylococcus aureus |
| MSSA | Methicillin-susceptible Staphylococcus aureus |
| NA | Not applicable |
| NICE | The National Institute for Health and Care Excellence |
| NIHR | National Institute for Health and Care Research |
| PHSI | Population Health Sciences Institute |
| SAEs | Serious Adverse Events |
| SAP | Statistical Analysis Plan |
| SAS | Safety analysis population |

**Contents**

[1. Introduction 5](#_Toc167277426)

[1.1 Background and rationale 5](#_Toc167277427)

[1.2 Hypotheses, objectives and outcome measures 5](#_Toc167277428)

[2. STUDY METHODS 8](#_Toc167277437)

[2.1 Trial design 8](#_Toc167277438)

[2.2 Framework 8](#_Toc167277439)

[2.3 Randomisation 8](#_Toc167277440)

[2.4 Sample size and power 8](#_Toc167277441)

[2.5 Interim analyses, data monitoring and stopping guidelines 9](#_Toc167277442)

[2.6 Timing of analyses 9](#_Toc167277443)

[2.7 Timing of outcome assessments 9](#_Toc167277444)

[3. Statistical principles 11](#_Toc167277445)

[3.1 Confidence intervals and p-values 11](#_Toc167277446)

[3.2 Analysis populations 11](#_Toc167277447)

[4. STUDY POPULATION 12](#_Toc167277497)

[4.1 Participant flow through trial 12](#_Toc167277498)

[4.1.1 Screening, eligibility and recruitment 13](#_Toc167277499)

[4.1.2 Withdrawals and availability of follow-up data 15](#_Toc167277500)

[4.2 Baseline characteristics 16](#_Toc167277501)

[4.3 Treatment adherence and protocol deviations 18](#_Toc167277502)

[4.3.1 Treatment adherence 18](#_Toc167277503)

[4.3.2 Protocol deviations 20](#_Toc167277504)

[5. analysIs methods 21](#_Toc167277505)

[5.1 Co-primary outcomes 21](#_Toc167277506)

[5.1.1 Definition of co-primary outcome measures 21](#_Toc167277507)

[5.1.2 Analysis methods 21](#_Toc167277508)

[5.1.3 Sensitivity analyses 22](#_Toc167277509)

[5.1.4 Supplementary analyses 22](#_Toc167277510)

[5.1.5 Subgroup analyses 23](#_Toc167277511)

[5.2 Secondary outcomes 24](#_Toc167277515)

[5.3 Exploratory outcomes 27](#_Toc167277516)

[5.4 Missing data 27](#_Toc167277517)

[6. SAFETY 28](#_Toc167277518)

[6.1 Adverse events 28](#_Toc167277519)

[6.2 Serious adverse events 28](#_Toc167277520)

[6.3 Other safety measures 28](#_Toc167277521)

[7. statistical software 29](#_Toc167277522)

[references 30](#_Toc167277523)

[APPENDIX 31](#_Toc167277524)

# **Introduction**

## Background and rationale

Sepsis is a syndrome of life-threatening organ dysfunction secondary to infection. It is a leading cause of death worldwide and a common reason for admission to a critical care unit, accounting for one third of general adult critical care admissions.

Antibiotics are initiated for the treatment of sepsis with guidelines recommending 7-10 days of antibiotic treatment, however the UK Health Security Agency and NICE both encourage the shortest duration of antibiotics needed. There is a lack of evidence to support antibiotic duration in sepsis, with guideline recommendations being largely extrapolated from non-sepsis populations. Furthermore, evidence to date suggests that clinicians are reluctant to discontinue antibiotics when presented with negative results. In practice, discontinuing antibiotics in critically ill patients is challenging since there are no accurate measures to inform clinicians that an infection has cleared. The minimum safe treatment duration required is unknown and it is likely that overtreatment occurs commonly. Determining whether a short, fixed-course of antibiotics is safe and effective in critically ill patients with sepsis is needed.

The SHORTER trial, funded by NIHR HTA, aims to determine the clinical and cost effectiveness of a short, fixed 5-day course of antibiotics compared to standard of care.

## Hypotheses, objectives and outcome measures

- - 1. **Trial hypotheses**

The trial is designed to separately test two null hypotheses:

- That mortality will be more than 6% higher in those randomised to a short, fixed 5-day initial antibiotic course at 28 days relative to those randomised to receive standard of care. The alternative hypothesis is that there is less than or equal to a 6% difference between randomised treatment groups (non-inferiority hypothesis).
- That there is no difference in the total number of antibiotic treatment days within 28 days of starting treatment, in those randomised to the receive a short, fixed 5-day initial antibiotic course compared to those randomised to receive standard of care. The alternative hypothesis is that there is a difference in antibiotic exposure at day 28, between randomised treatment groups (superiority hypothesis).
  - 1. **Primary objective and outcome measure**

The co-primary objectives and corresponding outcome measures are described in the table below. Detailed definitions of each co-primary outcome measure are provided in **section 5.1.1.**

|  | **Objectives** | **Outcome Measures** |
| --- | --- | --- |
| **Co-primary** | To determine whether short duration antibiotic therapy is non-inferior to standard of care in terms of mortality and reduces overall antibiotic exposure. | - 28-day all-cause mortality (non-inferiority safety outcome) - Total antibiotic treatment days measured at 28 days (superiority clinical effectiveness outcome) |

The co-primary clinical questions of interest are:

1. What is the absolute difference in the rate of 28-day all-cause mortality in adult patients admitted to a critical care unit with suspected or confirmed sepsis treated with an initial fixed 5-day antibiotic course compared to an antibiotic course determined as per standard of care, regardless of deviations from the allocated intervention for any reason or initiation of additional antibiotic courses? This estimand is described by the following attributes:

| Estimand attribute | Description of Estimand 1 |
| --- | --- |
| Population | Adult patients admitted to a critical care setting with antibiotics initiated for suspected or confirmed sepsis. The population outlined in Analysis Set 1 will be used for the analysis (See section 3.2 for more on analysis populations.) |
| Treatment conditions | Fixed 5-day initial antibiotic course (intervention)  Duration of initial antibiotic course as per standard of care (control) |
| Outcome measure | All-cause mortality 28-days from starting treatment – binary outcome |
| Strategies used to handle intercurrent events | - Deviation from fixed 5-day course for any reason – treatment policy - Receiving a 5-day course (or less) in the control arm – treatment policy - Initiation of additional antibiotic courses – treatment policy - Participants later found to be ineligible – treatment policy - Antibiotic clock restarted due to ineffective treatment – treatment policy |
| Population-level summary measure | Absolute difference (intervention – control) in the rate of 28-day all-cause mortality |

1. What is the absolute difference in the total number of antibiotic treatment days within 28 days of starting treatment? This estimand is described by the following attributes:

| Estimand attribute | Description of Estimand 2 |
| --- | --- |
| Population | Adult patients admitted to a critical care setting with antibiotics initiated for suspected or confirmed sepsis. The population outlined in Analysis Set 1 will be used for the analysis (See section 3.2 for more on analysis populations.) |
| Treatment conditions | Fixed 5-day initial antibiotic course (intervention)  Duration of initial antibiotic course as per standard of care (control) |
| Outcome measure | Total number of antibiotic treatment days within 28-days of starting treatment |
| Strategies used to handle intercurrent events | - Death – while alive; zero antibiotic days assumed following death - Participant found to be ineligible – treatment policy |
| Population-level summary measure | Mean difference (intervention – control) in 28-day antibiotic treatment days |

- - 1. **Secondary objectives and outcome measures**

The secondary objectives and corresponding outcome measures are described in the table below. More detailed definitions of each secondary outcome measure are provided in **Section 5.2.1.**

|  | **Objectives** | **Outcome Measures** |
| --- | --- | --- |
| To assess the effect of short duration antibiotic therapy on: | | |
| 1. | 90-day mortality | All-cause mortality at 90 days |
| 2. | Suspected clinically relevant antibiotic-associated adverse events | Suspected clinically relevant antibiotic-associated adverse events during index hospital admission occurring from randomisation up to discharge |
| 3. | Days alive and out of hospital up to 90 days | Number of days alive and out of hospital from discharge to day 90, with a penalty for death |
| 4. | Length of critical care unit stay | Duration (number of days) of critical care unit stay up to 90 days |
| 5. | Length of hospital-stay | Duration (number of days) of hospital-stay up to 90 days |
| 6. | Duration of initial antibiotic course for sepsis | Duration (number of days) of Initial antibiotic treatment |
| 7. | Rate of further/recurrence of infections | Number of further/recurrence of infections requiring additional antibiotic courses following index sepsis episode up to 28 days |
| 8. | Readmission to critical care or hospital | Occurrence of readmission to critical care or hospital during the 90 day follow up period |

For outcomes where death would be considered an intercurrent event a ‘while alive’ strategy will primarily be used.

- - 1. **Exploratory objectives and outcome measures**

There are no planned exploratory objectives or outcome measures for this trial.

# **STUDY METHODS**

## Trial design

SHORTER is a pragmatic, open-label, phase III, multi-centre, parallel arm, randomised controlled trial with co-primary non-inferiority and superiority outcomes. Eligible and consenting adult patients with suspected or confirmed sepsis requiring admission to a critical care unit (including both high dependency units and intensive care units) will be randomised in a 1:1 ratio to receive a short, fixed 5-day initial course of antibiotic treatment or standard of care.

## Framework

The co-primary outcomes of this trial are 28-day-all-cause mortality and total antibiotic treatment days measured at 28 days following treatment initiation.

The first primary outcome measure is to be compared under a non-inferiority hypothesis testing framework (to determine whether a short, fixed 5-day initial course of antibiotic treatment is non-inferior to an antibiotic course delivered by standard of care). The second primary outcome measure will be tested for superiority, determining whether those assigned to the short course have a reduced number of total antibiotic treatment days compared to those assigned to standard of care within 28 days of starting treatment. All analyses will be conducted using a frequentist approach, unless otherwise stated.

Where secondary outcomes are formally compared, these will also be tested for superiority.

## Randomisation

Random permuted blocks of variable length will be used to allocate participants in a 1:1 ratio to either a short, fixed 5-day initial course of antibiotic treatment or standard of care. Randomisation is stratified by centre (with approximately 50 participating sites) and infection type (either community-acquired or hospital-acquired infection). Detail of the block length(s) used is held in a restricted location in the electronic study folder and will not be disclosed until after final data lock and analysis.

## Sample size and power

The recruitment target for this trial is a total of 2244 patients, 1122 in each treatment group.

The sample size is based on assessing non-inferiority for 28-day all-cause mortality. Assuming a 28-day mortality of 24% [1], a sample size of 1065 patients per arm will have 90% power, with a one-sided alpha of 2.5%, to demonstrate non-inferiority with a margin of 6%. Allowing for a 5% drop out rate, the total sample size will be 2244 patients. The sample size will also have 90% power to detect a 1.1-day difference in total antibiotic treatment days to 28 days, assuming a standard deviation of 7.7 (based on ADAPT-sepsis trial data) and a two-sided alpha of 5%.

Determining non-inferiority margins for antibiotic trials is a challenge due to the lack of historical placebo-controlled trials. The Food and Drug Administration (FDA) recommends a 10% non-inferiority margin for mortality outcomes in community acquired pneumonia trials [2]. In addition, recommended non-inferiority margins for healthcare-associated pneumonia trials range from 7-10% [3,4]. Previous trials of antibiotic durations for ventilator associated pneumonia [5] and biomarker-guided antibiotic durations for critically ill patients with sepsis have used non-inferiority margins of 8-10% for mortality outcomes [6,7]. We selected a non-inferiority of 6% margin which will be one of the lowest used in antibiotic duration trials.

This sample size was determined based on a two-group test of non-inferiority in proportions performed using the proprietary software nQuery.

## Interim analyses, data monitoring and stopping guidelines

There are no formal interim analyses planned for this trial, however the trial data will be monitored by an Independent Data Monitoring Committee (IDMC) that will meet at the start of the trial and at approximately 6 monthly intervals thereafter, unless otherwise agreed. As per the IDMC charter, the IDMC will review accumulating data on recruitment, data quality, adherence to treatment, follow-up, safety, and key co-primary and secondary outcome measures. Underlying assumptions pertaining to the sample size calculations, including overall numbers of deaths, will also be monitored by the IDMC.

The IDMC will be unblinded to treatment allocation, with descriptive summaries of data presented by randomised treatment group in closed reports. Three statisticians will be involved in the trial; one will remain blinded to treatment allocation until the final data lock for analysis and two will be unblinded and will be responsible for the preparation and review of interim closed reports to the IDMC. The unblinded statisticians will also be responsible for performing the final analysis.

An internal pilot will be conducted for the first 12 months of recruitment to assess site opening, recruitment rates and group separation in antibiotic treatment days. The following progression criteria will be used to assess progression to the full trial at 12 months:

| **Progression criteria** | **Red** | **Amber** | **Green** |
| --- | --- | --- | --- |
| Trial recruitment 30% complete (i.e. 673 recruited) | <50% | ≥50%, <100% | 100% |
| Recruitment rate/site/month | <1/site/month | 1-2/site/month | 2/site/month |
| Number of sites open | <25 | ≥25, <50 | 50 |
| Between group difference (control - intervention) in duration of initial antibiotic course (intervention period; antibiotic days) | <1 | ≥1, <2 | ≥2 |

## Timing of analyses

The final analysis will take place once the last participants final follow up is complete, i.e., the final participant reaches day 90 follow up time point, and the associated forms are complete. Once all data queries have been resolved (as far as possible) the database will be locked and the final analysis will commence.

## Timing of outcome assessments

Trial days will be counted in 24 hour periods, following the start of antibiotic initiation for suspected or confirmed sepsis. Trial day 1 marks the first 24-hour period following antibiotic initiation for sepsis. All trial intervention and outcome days will be counted from this start point, with subsequent days counted in consecutive 24-hour periods. Participants are followed up for a total of 90 days.

The outcomes assessed at each trial day is specified in the table below.

**Schedule of events**

| **Trial day** | **Day 1** | **Day 2** | **Day 3** | **Day 4** | **Day 5** | **Day 6** | **Day 7** | **Day 28** | **Discharge** | **Day 90** |
| --- | --- | --- | --- | --- | --- | --- | --- | --- | --- | --- |
| Screening, Consent, Randomisation | X | | | |  |  |  |  |  |  |
| Baseline data collection | X | | | |  |  |  |  |  |  |
| Laboratory data collection | X |  | X |  | X |  | X |  | X |  |
| SOFA score | X |  |  |  | X |  | X |  |  |  |
| Functional Comorbidity Index | X |  |  |  |  |  |  |  |  |  |
| APACHE II Score | Within 24 hours of admission to critical care | | | |  |  |  |  |  |  |
| Assessment of duration of initial course |  |  |  |  | X | | | |  |  |
| All-cause mortality assessment |  |  |  |  |  |  |  | X |  | X |
| Antibiotic treatment days to 28 days |  |  |  |  |  |  |  | X |  |  |
| Adverse event reporting | Reported from randomisation up to discharge | | | | | | | | |  |
| Length of critical care unit stay |  |  |  |  |  |  |  |  | X |  |
| Length of hospital stay |  |  |  |  |  |  |  |  | X |  |
| Further/reoccurrence of infection requiring antibiotic courses |  |  |  |  |  |  |  | X |  |  |
| Readmission to hospital |  |  |  |  |  |  |  |  |  | X |
| Readmission to critical care |  |  |  |  |  |  |  |  |  | X |

# **Statistical principles**

## Confidence intervals and p-values

This trial has two co-primary objectives / outcome measures:

1. To determine whether short duration antibiotic therapy is non-inferior to standard of care in terms of 28-day all-cause mortality
2. To determine whether a short, fixed course of antibiotic treatment reduces overall antibiotic exposure, compared to patients receiving standard of care

For each co-primary outcome, a different level of statistical significance will be used.

For the analysis of the first co-primary outcome, 28-day all-cause mortality, a one-sided hypothesis test will be performed and interpreted using a significance level of 0.025. Confidence intervals will be two-sided and reported at the 95% level. Non-inferiority will be demonstrated if the upper limit of the 95% confidence interval is below the non-inferiority margin of 6%.

For the analysis of the second co-primary outcome, total days of antibiotic exposure within 28 days of treatment initiation, a two-sided hypothesis will be performed and interpreted using a significance level of 0.05. Confidence intervals will also be two-sided and reported at the 95% level.

Confidence intervals and p-values will be provided for key secondary outcomes; however these will be considered exploratory in nature and therefore no adjustment for multiple testing has been made.

## Analysis populations

The following analysis sets will be defined:

| Analysis Set 1 (AS1) | All randomised participants with outcome data available* will be included in the analysis and analysed according to the treatment group they were randomised to receive, i.e. following the intention-to-treat (ITT) principle |
| --- | --- |
| Analysis Set 2 (AS2) | As above (AS1), but participants found to be ineligible after randomisation will be excluded |
| Analysis Set 3 (AS3) | As above (AS1), but participants found to be ineligible after randomisation and participants deviating from the short course in error will be excluded |
| Safety Analysis Set (SAS) | All randomised participants analysed according to the treatment group they were randomised to receive, i.e. following the intention-to-treat (ITT) principle |

*defined for each outcome in Section 5

Safety data (adverse events) will be analysed and reported using the SAS. For all other outcomes AS1 will be used for the main analyses. AS2 and AS3 will be used to perform supplementary analyses of the co-primary outcomes.

# **STUDY POPULATION**

## Participant flow through trial

Patient flow through the trial will be presented using a CONSORT diagram, see example below. Information will be provided on numbers and reasons (where appropriate) for: screened patients not being eligible; eligible patients not being randomised; participants not receiving short course treatment in error; withdrawal from follow-up; patients not evaluable for the primary endpoints. The number of participants remaining in the study at days 7, 28, and 90 will also be shown.

**Example CONSORT flow diagram**

**
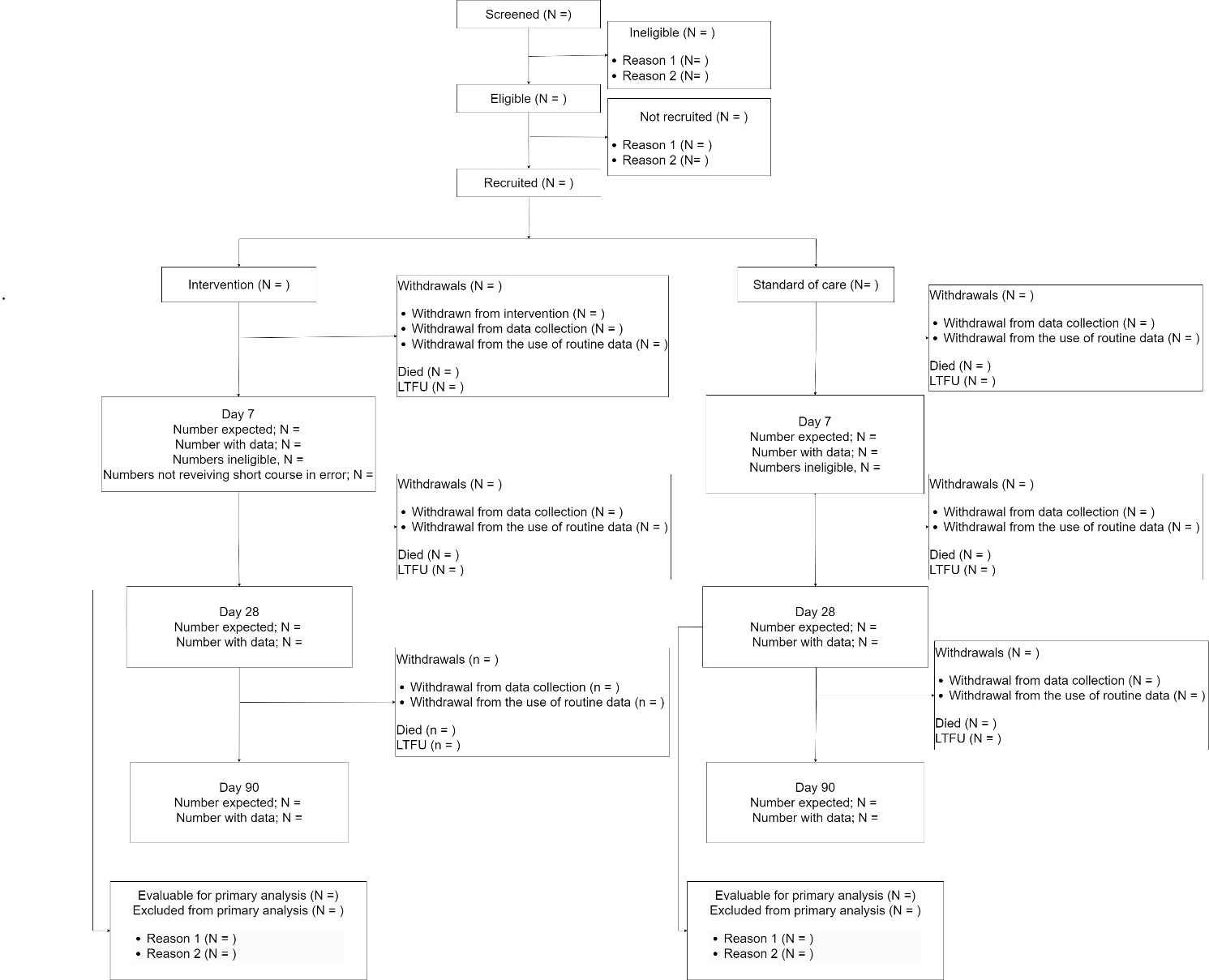
**

## Screening, eligibility and recruitment

The following screening data will be provided to describe the representativeness of the study sample:

- Number of participants screened for eligibility, by site and overall
- The number and proportion (of those screened) found to be eligible, by site and overall
- The number and proportion (of those eligible) recruited, by site and overall
- The number and proportion (of those recruited) with capacity at screening, by site and overall
- Reasons for ineligibility, reported as frequency and percentage (of those ineligible), by site and overall.
- Reasons for eligible patients not taking part, reported as frequency and percentage (of those eligible and not taking part), overall and by person deciding on participation (patient or relative / welfare guardian).

Final recruitment will be presented graphically as the cumulative recruitment rate over time, see **Figure 1** as an example. Projected recruitment rates may also be shown, for reference.

**Example Table 1: Summary of screening and accrual data by site.**

| Site | Months open to recruitment | Screened | Eligible | Recruited | With capacity |
| --- | --- | --- | --- | --- | --- |
|  |  |  | N (%^1^) | N (%^2^) | N (%^3^) |
| Newcastle |  |  |  |  |  |
| King’s College |  |  |  |  |  |
| Ipswich |  |  |  |  |  |
| Royal Papworth |  |  |  |  |  |
| Sunderland |  |  |  |  |  |
| Northumbria |  |  |  |  |  |
| Total |  |  |  |  |  |

*^1^Of those screened.*

*^2^Of those eligible.*

*^3^Of those recruited.*

**Sites ordered by total number recruited.*

**Example Table 2. Reasons for ineligibility***

| Reason | N (%) |
| --- | --- |
| Is > 18 years of age |  |
| Participant is not being treated within a critical care setting (ICU or HDU) for suspected or confirmed sepsis due to either community- or hospital-acquired infection |  |
| No evidence of new or worsening acute organ dysfunction resulting from suspected or confirmed infection (i.e. no treatment or monitoring of organ function) |  |
| No initiation of antibiotics for suspected or confirmed sepsis |  |
| Patient not able to be randomised within 4 days of the initiation of antibiotics for the treatment of suspected or confirmed sepsis |  |
| Comorbidity with immunosuppression (e.g., chemotherapy, maintenance steroids equivalent to >10 mg/day of prednisolone, post-transplantation) |  |
| Blood neutrophil count less than 0.5 x 10^9^/L secondary to a pre-existing comorbidity |  |
| Infection source where usual practice involves more than 14 days of antibiotics (e.g. undrainable abscess, endocarditis, Staphylococcus aureus bacteraemia, osteomyelitis) |  |
| Receiving end-of-life care |  |
| Life-sustaining treatment expected to be withdrawn in the next 24 hours |  |
| The clinician responsible for the patient's care is unable to adhere to the intervention |  |
| Total |  |

**Will also be reported by site*

**Example Table 3. Reasons for eligible patients not participating.**

|  | Decision made by | | Overall |
| --- | --- | --- | --- |
|  | Patient | Welfare guardian/relative |  |
|  | N (%) | N (%)* | N (%) |
| Not willing to be randomised to a short course of antibiotics |  |  |  |
| Does not want to take part in research |  |  |  |
| Does not like the trial design/trial design too onerous |  |  |  |
| Not willing to share personal data |  |  |  |
| Sees no benefit in the trial |  |  |  |
| No reason given |  |  |  |

**For those without capacity at screening*

**Example Figure 1. Final observed accrual**

***
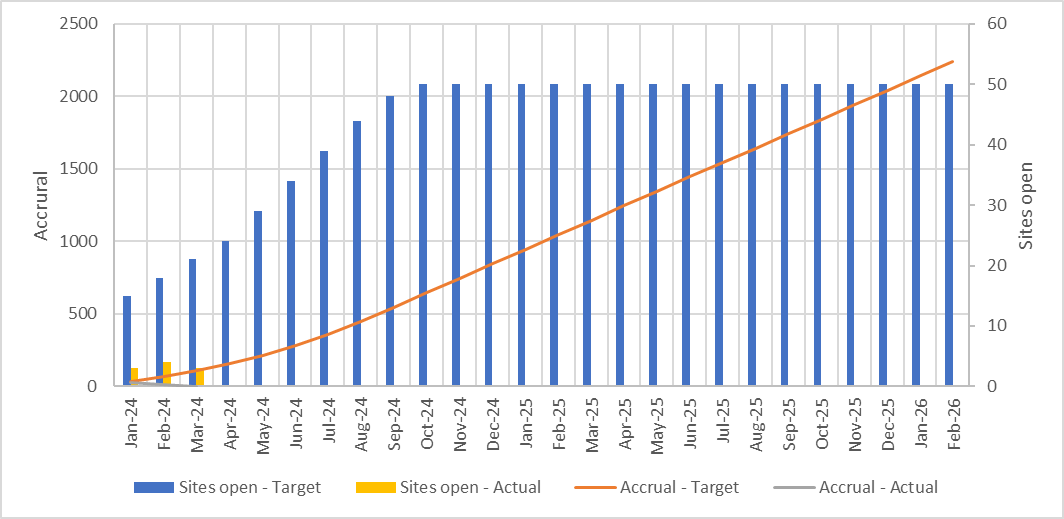
***

## Withdrawals and availability of follow-up data

In this trial patients may withdraw from:

1. Trial intervention
2. Follow up data collection
3. Use of routine data

The number and proportion of participants no longer available for follow-up will be summarised at specific time points: before day 7, before day 28 and before day 90. The numbers that died, were lost to follow-up (LTFU) or withdrew from follow-up (and the type of withdrawal) will be tabulated as frequency and percentage in each randomised group, see example **Table 4**. Reasons for withdrawals, and whether this was participant or investigator led will also be tabulated by randomised treatment group, see example **Table 5**.

**Example Table 4: Summary of withdrawals, deaths and numbers LTFU, presented by arm.**

|  | Short course | Standard of Care | Overall |
| --- | --- | --- | --- |
|  | N = | N = | N = |
| Before day 7 |  |  |  |
| Withdrawal from^1^: |  |  |  |
| Trial intervention |  |  |  |
| Follow up data collection |  |  |  |
| The use of routine data |  |  |  |
| All three |  |  |  |
| Died |  |  |  |
| LTFU |  |  |  |
| Before day 28 |  |  |  |
| Withdrawal from^1^: |  |  |  |
| Follow up data collection |  |  |  |
| The use of routine data |  |  |  |
| Both |  |  |  |
| Died |  |  |  |
| LTFU |  |  |  |
| Before day 90 |  |  |  |
| Withdrawal from^1^: |  |  |  |
| Follow up data collection |  |  |  |
| The use of routine data |  |  |  |
| Both |  |  |  |
| Died |  |  |  |
| LTFU |  |  |  |

**Data are N(%)*

*^1^Options are not mutually exclusive.*

**Example Table 5. Reasons for withdrawal, presented by randomised treatment group.**

|  | Short course | Standard of Care | Overall |
| --- | --- | --- | --- |
|  | N = | N = | N = |
| Investigator led withdrawal | N = | N = | N = |
| Participant lost capacity for a prolonged period and cannot comply with trial procedures |  |  |  |
| AE which resulted in their inability to continue to comply with trial procedures |  |  |  |
| Investigator deems it inappropriate for the participant to continue with the trial |  |  |  |
| Participant led withdrawal | N = | N = | N = |
| Burden of assessments |  |  |  |
| Personal circumstances |  |  |  |
| After recovering capacity patient was not interested in taking part |  |  |  |

## Baseline characteristics

Baseline characteristics will be summarised descriptively, both overall and by randomised treatment group. Categorical variables will be summarised by frequency and percentage. Continuous data will be summarised by the mean and standard deviation and/or median, IQR and range, as appropriate. No significance testing will be carried out due to the randomised nature of the study.

Baseline characteristics will primarily be reported in all randomised participants; however data may also be presented in additional analysis populations (as defined in section 3.2) to assess whether any exclusions may introduce selection bias.

Details of characteristics to be reported are given in the example **Table 6 and Table 7** below.

**Example Table 6: Baseline characteristics by randomised treatment group.**

|  | Short course | Standard of Care | Overall |
| --- | --- | --- | --- |
|  | (N = ) | (N = ) | (N = ) |
| Age (years) |  |  |  |
| Mean (SD) |  |  |  |
| Min, Max |  |  |  |
| <40 |  |  |  |
| 40-65 |  |  |  |
| 66-80 |  |  |  |
| >80 |  |  |  |
| Sex |  |  |  |
| Female |  |  |  |
| Male |  |  |  |
| Ethnicity |  |  |  |
| White – UK |  |  |  |
| White - Roma |  |  |  |
| White - Gypsy or Irish Traveller |  |  |  |
| Black - Caribbean |  |  |  |
| Black - African |  |  |  |
| Asian - Indian |  |  |  |
| Asian - Pakistaini |  |  |  |
| Asian - Bangladeshi |  |  |  |
| Asian - Chinese |  |  |  |
| Mixed - White & Black Caribbean |  |  |  |
| Mixed - White & Black African |  |  |  |
| Mixed - White & Asian |  |  |  |
| Arab |  |  |  |
| APACHE II |  |  |  |
| Mean (SD) |  |  |  |
| Median (IQR); Range |  |  |  |
| Functional Comorbidity index |  |  |  |
| Mean (SD) |  |  |  |
| Median (IQR); Range |  |  |  |
| SOFA score |  |  |  |
| Mean (SD) |  |  |  |
| Median (IQR); Range |  |  |  |
| Critical care admission |  |  |  |
| Admission type to critical care |  |  |  |
| Medical |  |  |  |
| Surgical |  |  |  |
| *Category of admission:* |  |  |  |
| Respiratory |  |  |  |
| Gastrointestinal or liver |  |  |  |
| Cardiovascular |  |  |  |
| Trauma |  |  |  |
| Obstetrics and gynaecology |  |  |  |
| Neurological (non-trauma) |  |  |  |
| Days from hospital admission to critical care admission: |  |  |  |
| Median (IQR); range |  |  |  |
| Days from critical care admission to randomisation: |  |  |  |
| Median (IQR); range |  |  |  |
| Support received |  |  |  |
| Organ support |  |  |  |
| Renal replacement therapy |  |  |  |
| Respiratory support |  |  |  |
| Blood pressure support |  |  |  |
| Corticosteroids |  |  |  |
| Within 24 hours of sepsis |  |  |  |
| Highest temperature (°C) |  |  |  |
| Mean (SD) |  |  |  |
| Median (IQR); Range |  |  |  |
| Lactate (mmol/L) |  |  |  |
| Mean (SD) |  |  |  |
| Median (IQR); Range |  |  |  |

**Example Table 7. Infection source causing sepsis, by randomised treatment group.**

|  | Short course | Standard of Care | Overall |
| --- | --- | --- | --- |
|  | (N = ) | (N = ) | (N = ) |
| Where did the patient develop sepsis? |  |  |  |
| Community acquired |  |  |  |
| Hospital acquired |  |  |  |
| Primary site of infection causing sepsis |  |  |  |
| Respiratory tract |  |  |  |
| Central nervous system |  |  |  |
| Skin and soft tissue |  |  |  |
| Central line related infection |  |  |  |
| Intra-abdominal |  |  |  |
| Urinary tract |  |  |  |
| Ear nose and throat |  |  |  |
| Blood stream |  |  |  |
| Unknown focus |  |  |  |
| Secondary site of infection causing sepsis |  |  |  |
| No secondary site |  |  |  |
| Respiratory tract |  |  |  |
| Central nervous system |  |  |  |
| Skin and soft tissue |  |  |  |
| Central line related infection |  |  |  |
| Intra-abdominal |  |  |  |
| Urinary tract |  |  |  |
| Ear nose and throat |  |  |  |
| Blood stream |  |  |  |
| Unknown focus |  |  |  |
| Causative microorganism identified for the infection?* |  |  |  |
| Yes |  |  |  |
| No |  |  |  |

**Further details on the specific types of causative microorganisms can be found in the* ***Appendix.***

## Treatment adherence and protocol deviations

## Treatment adherence

In this trial, participants are randomised to receive either a short, fixed 5-day course of antibiotics or standard of care. The intervention concerns only the initial course of antibiotic treatment for sepsis; care is taken to differentiate an initial and further course of treatment.

The initial course of antibiotic treatment for sepsis will be measured from the date and time antibiotics were initiated to the date and time the initial course of antibiotic treatment for sepsis was stopped. The difference between these two timepoints will be calculated in hours and divided by 24 to give the duration of the initial antibiotic course in 24-hour periods (full days).

There may be changes to the dose, frequency, or antibiotic class prescribed. Treatment may further stop and restart. Alterations to prescribed treatment is considered an extension of the initial course. Breaks in antibiotic administration less than or equal to 24-hours are also considered a continuous extension of the initial course. Breaks in antibiotic administration > 24 hours however will constitute a new, further course of treatment and will not be counted towards the initial course.

See below examples of two antibiotic treatment courses where treatment has spanned 7 days, but the initial antibiotic course is classified differently. **Example 1** shows the scenario where antibiotics are stopped and restarted, with a break ≤ 24 hours, prolonging the initial course. While in **Example 2** antibiotics are ceased for > 24 hours, ending the initial course of treatment.

**Example 1: Antibiotic course restarted within 24h**

| Day | Mon | Tues | Wed | Thurs | Fri | Sat | Sun |
| --- | --- | --- | --- | --- | --- | --- | --- |
| Example drug chart | **Day 1** | **Day 2** | **Day 3** | **Day 4** | **Day 5** | **Day 6** | **Day 7** |
|  | 6am ❖ | 6am ✓ | 6am ✓ | 6am ✓ | 6am ✓ | 6am | 6am ✓ |
|  | 2pm ✓ | 2pm ✓🞟 | 2pm ✓ | 2pm ✓ | 2pm 🔾 | 2pm ❖ | 2pm ✓ |
|  | 10pm✓ | 10pm ✓ | 10pm ✓ | 10pm ✓ | 10pm | 10pm ✓ | 10pm🔾 |
|  | Initial course between Mon 6am – Sun 10pm, 160 hours between each timepoint. | | | | | | |
| Calculation | Full days on treatment: 160/24 = 6.67 | | | | | | |
|  | Initial antibiotic course: 6.67 days | | | | | | |

**Treatment stopped on Friday, but restarted ≤24 hours later, extending the initial course of treatment.*

*❖Treatment for sepsis initiated, first dose taken; 🞟Changed dose; ✓Antibiotic dose taken; 🔾Final dose taken, treatment stopped.*

**Example 2: Antibiotic course restarted with > 24h break in treatment**

| Day | Mon | Tues | Wed | Thurs | Fri | Sat | Sun |
| --- | --- | --- | --- | --- | --- | --- | --- |
| Example drug chart | **Day 1** | **Day 2** | **Day 3** | **Day 4** | **Day 5** | **Day 6** | **Day 7** |
|  | 6am ❖ | 6am ✓ | 6am ✓ | 6am | 6am ❖ | 6am✓ | 6am ✓ |
|  | 2pm ✓ | 2pm ✓ | 2pm ✓ | 2pm | 2pm | 2pm✓ | 2pm 🔾 |
|  | 10pm ✓*🞟* | 10pm ✓ | 10pm 🔾 | 10pm | 10pm✓ | 10pm ✓ | 10pm |
| Calculation | Initial course between Mon 6am - Wed 10pm, 64 hours between each timepoint. | | |  | Further course between Fri 6am - Sun 2pm, 56 hours between each timepoint. | | |
|  | Full days on treatment: 64/24 = 2.67 | | |  | Full days on treatment: 56/24 = 2.33 | | |
|  | Initial antibiotic course: 2.67 days | | |  | Further course: 2.33 days | | |

**Treatment stopped on Wednesday, but restarted >24 hours later, ending the initial course on Wednesday, and starting a further course of treatment on Friday.*

*❖Treatment for sepsis initiated, first dose taken; 🞟Changed dose; ✓Antibiotic dose taken; 🔾Final dose taken, treatment stopped.*

The duration of the initial antibiotic treatment course will be summarised descriptively, in days, using the mean and standard deviation and/or median, IQR and range, by randomised treatment. Also presented will be the number and proportion of participants receiving less than 5 days of treatment, 5 days of treatment, or more than 5 days. Where the intervention (fixed 5-day course) was not adhered to, reasons why are presented. Where the trial intervention was not adhered to in error, this will form a protocol deviation. See **Table 8** as an example.

Patients with blood culture proven resistance to the initial antibiotic treatment can have their ‘clock reset’ for the initial course of antibiotics, once adequate antibiotics have been commenced. The duration of the initial course will be measured from this ‘reset’ date and time. The number and proportion of participants with their Trial “Day 1” reset due to ineffective treatment will be presented, along with the numbers occurring before or after randomisation. The duration of the initial course prior to clock reset will be summarised using the mean and standard deviation and/or median, IQR and range, by randomised treatment. See **Table 9** as an example.

**Example Table 8: Summary of initial antibiotic treatment received.**

|  | Short course | Standard of Care | Overall |
| --- | --- | --- | --- |
|  | N = | N = | N = |
| Duration of initial antibiotic course |  |  |  |
| Mean (SD) |  |  |  |
| Median (IQR); range |  |  |  |
| Duration of initial antibiotic course |  |  |  |
| < 5-day course |  |  |  |
| Fixed 5-day course |  |  |  |
| > 5-day course |  |  |  |
| Reasons for patients not adhering to short course^1^ |  |  |  |
| Patient withdrew from trial intervention |  | NA |  |
| Clinical decision |  | NA |  |
| Error |  | NA |  |
| No reason given |  | NA |  |

*^1^ Intervention arm only*

**Example Table 9: Ineffective treatment and clock reset.**

|  | Short course | Standard of Care | Overall |
| --- | --- | --- | --- |
|  | N = | N = | N = |
| Clock reset | N (%) | N (%) | N (%) |
| When was the clock reset? |  |  |  |
| Before randomisation |  |  |  |
| Post randomisation |  |  |  |
| Duration* of initial course prior to reset |  |  |  |
| Mean (SD) |  |  |  |
| Median (IQR); range |  |  |  |

**Days*

## Protocol deviations

Clinicians are encouraged but are not mandated to adhere to a fixed 5-day course of antibiotic treatment and are able to use clinical discretion to determine the course of treatment for a patient. Intentional non-adherence therefore is permitted. Non-adherence due to an error however will be considered a protocol deviation. Protocol deviations will be provided in a line listing or tabulated by randomised treatment group, depending on the number reported.

Should any major protocol deviations be reported during the trial which have not already been considered in the statistical analysis plan, a decision on whether the participant should be included/excluded from analysis populations will be reviewed and agreed by a statistician without unblinded access to study outcome data.

**Example Table 10. Protocol deviations**

| ID | Site | Treatment group | Time (in days) from randomisation to deviation | Deviation type* | Major or minor deviation | Details |
| --- | --- | --- | --- | --- | --- | --- |
|  |  |  |  |  |  |  |
|  |  |  |  |  |  |  |

**A. Consent Procedures, B. Eligibility Criteria/ Confirmation, C. SAE Reporting, D. Randomisation Procedures, E. Intervention Management, F. Trial Procedures, G. Site Staff, H. Training, I. Source Data/Source Data Collection, J. Participant Confidentiality/Data Protection, K. GCP, L. PI Oversight, M. Other*

# **analysIs methods**

## Co-primary outcomes

## Definition of co-primary outcome measures

**28-day mortality**

The 28-day timepoint will be measured from the start of the antibiotic course for sepsis, defined as ‘trial day 1’. The index course of antibiotics are the antibiotics the participant is receiving for sepsis at the point of randomisation. ‘Trial day 1’ is the start of that index course of antibiotics. This start date is likely to be a pre-randomisation event for most participants.

However, if the initial course of antibiotics for sepsis proves to be inadequate due to a blood culture-proven resistant organism and antibiotics are changed, then ‘trial day 1’ will be reset with the date of starting an adequate treatment course of antibiotics. The 28-day timepoint will then be measured from the ‘reset’ trial day 1.

28-day mortality will be defined as death due to any cause within 28 days of ‘trial day 1’. Any participant who withdraws from trial follow-up and from providing routinely collected data prior to day-28 will not be included in the analysis as their survival status at day-28 will be unknown.

**28-day antibiotic days**

The 28-day timepoint will be defined as above.

28-day antibiotic days will be defined as the number of calendar days within 28 days of ‘trial day 1’ where antibiotic treatment was taken. Only antibiotics for the active treatment of an infection will count towards this outcome.

As above, any participant who withdraws from trial follow-up and from providing routinely collected data prior to day-28 will not be included in analysis.

## Analysis methods

The attributes of each estimand are described in **section 1.1.2.**

**28-day mortality**

Analyses will be performed using AS1, see section 3.2, following the ITT principle. Participants who withdraw from trial follow-up and from providing routinely available data prior to day-28 will not be included in the analysis. This analysis maps to Estimand 1.

The number and proportion of participants who died within 28-days will be reported descriptively by randomised treatment group. The treatment effect will be estimated as an absolute difference in proportions (intervention – control) by fitting a binomial generalised estimating equation model with an identity link function to the outcome data. To allow for centre-effects an exchangeable correlation structure will be used. This approach follows recommended methods [8]. The model will also be adjusted for community-acquired versus hospital-acquired infection, which is a stratification factor for the randomisation process. The treatment effect estimate will be reported with a 95% confidence interval. Non-inferiority will be demonstrated if the upper limit of the 95% confidence interval is below the non-inferiority margin of 6%. A one-sided p-value for non-inferiority (with non-inferiority margin of 6%) will be calculated using a Wald test with a critical threshold of 0.025.

Should the planned analysis model fail to converge alternative methods will be used, such as a mixed effects generalised linear model, or using ordinary least squares estimates rather than maximum likelihood estimation [9].

Model diagnostics, goodness of fit and influential observations will be examined.

**28-day antibiotic days**

Analyses will be performed using AS1, see section 3.2, following the ITT principle. This analysis maps to Estimand 2.

28-day antibiotic days will be summarised descriptively by randomised treatment group using the mean and standard deviation and/or median, IQR and range. The treatment effect will be estimated as the mean difference (intervention - control) by fitting a mixed-effects linear regression model, with centre included as a random effect and community-acquired versus hospital-acquired infection as a fixed effect. The treatment effect estimate will be reported with a 95% confidence interval and a two-sided p-value for superiority will be calculated using a Wald test with a critical threshold of 0.05.

The assumptions of the model will be explored, for example via plots of residuals versus fitted values and normal quantile plots of standardised residuals.

## Sensitivity analyses

If the proportion of participants who are excluded from analyses due to withdrawal from trial follow-up and from providing routinely available data prior to day-28 is greater than 5%, either overall or in either treatment group, sensitivity analyses using imputation methods will be explored. More detail is provided in section 5.4.

## Supplementary analyses

**28-day mortality**

The following supplementary analyses will be performed:

- The relative difference in 28-day mortality between randomised treatment groups (intervention/control) will be estimated using a suitable mixed-effects generalised linear model with centre included as a random effect and other stratification factors as fixed effects. Analyses will be performed using AS1, as above.
- Analyses described in section 5.1.2 will be repeated but using AS2, which will exclude participants who were found to be ineligible after randomisation.
- Analyses described in section 5.1.2 will be repeated but using AS3, which will exclude participants who were found to be ineligible after randomisation and who did not receive short course treatment in error.
- Analyses described in section 5.1.2 will be repeated but using a hypothetical strategy to estimate the treatment effect if no participants had withdrawn from trial treatment
- Views on an acceptable non-inferiority margin may vary by stakeholder. To address this, we will utilise a Bayesian multilevel binomial generalised linear model to calculate posterior probabilities that short duration treatment is non-inferior to standard of care for a range of non-inferiority thresholds up to 6%. We will use an uninformative prior if there is no suitable quality evidence available. The results will be presented graphically as non-inferiority acceptability curves.

**28-day antibiotic days**

The following supplementary analyses will be performed:

- The relative difference in 28-day antibiotic days between randomised treatment groups (intervention/control) will be estimated using a suitable mixed-effects generalised linear model with centre included as a random effect and other stratification factors as fixed effects. Analyses will be performed using AS1, as above.
- Analyses described in section 5.1.2 will be repeated but using AS2, which will exclude participants who were found to be ineligible after randomisation.
- Analyses described in section 5.1.2 will be repeated but using AS3, which will exclude participants who were found to be ineligible after randomisation and who did not receive short course treatment in error.
- Analyses described in section 5.1.2 will be repeated but using a hypothetical strategy to estimate the treatment effect if no participants chose to withdraw from trial treatment
- Causal effect models will be used to estimate the Survivor Average Causal Effect (SACE) if there are differences in 28-day mortality rates between treatment groups. This will estimate the treatment effect in the sub-population of participants who would have survived to day 28 regardless of treatment allocation.

## Subgroup analyses

We will explore whether the treatment effect for each of the co-primary outcomes are consistent across subgroups. To do this will fit models (as described in section 5.1.2) with an interaction between treatment and the subgroup variable of interest.

For each subgroup, the estimated treatment effect and 95% CI will be presented using Forest plots which will also show the p-value from a test of interaction. The following sub-groups will be explored:

- Culture-negative sepsis. Defined as no positive culture results from samples taken in relation to index course of antibiotics for sepsis.
- Positive blood culture. Defined as a positive blood culture from a sample taken in relation to index course of antibiotics for sepsis.
- Site of infection including community acquired pneumonia, urinary tract infection and intra-abdominal infection
- Septic shock status
- Baseline severity of illness
- Blood culture proven resistance to the initial antibiotic treatment requiring the initial antibiotic treatment course to be reset (for mortality co-primary outcome only)

As the trial has not been powered to detect subgroup effects these results will be considered exploratory and hypothesis generating. Conclusions will not be drawn on the basis of these results.

## Secondary outcomes

**90-day all-cause mortality**

90-day mortality will be defined as death due to any cause within 90 days of ‘trial day 1’, as defined in section 5.1.1.

Analyses will be performed using AS1, see section 3.2, following the ITT principle. Participants who withdraw from trial follow-up and from providing routinely available data prior to day-90 will not be included in the analysis as their survival status at day-90 will be unknown.

The number and proportion of participants who died within 90-days will be reported descriptively by randomised treatment group. The treatment effect will be estimated as an absolute difference in proportions (intervention – control) by fitting a binomial generalised estimating equation model with an identity link function to the outcome data. To allow for centre-effects an exchangeable correlation structure will be used. The model will also be adjusted for community-acquired versus hospital-acquired infection, which is a stratification factor for the randomisation process. The treatment effect estimate will be reported with a 95% confidence interval.

**Time to death**

Time will be measured, in days, from ‘trial day 1’, as defined in section 5.1.1, to date of death from any cause. Participants who are alive will be censored at the date they were last known to be alive, which will be the latest date of 1) date patient status was checked at Day 90, 2) date patient status was checked at Day 28, or 3) date of withdrawal.

Analyses will be performed using AS1, see section 3.2, following the ITT principle.

Event rates will be estimated over time using Kaplan-Meier methods and will be presented using Kaplan-Meier survival curves. The treatment effect will be estimated using the Hazard Ratio (HR), estimated by fitting a Cox proportional hazards regression model. To allow for centre-effects a shared-frailty term for recruiting site will be included. The model will also be adjusted for community-acquired versus hospital-acquired infection. The treatment effect estimate will be reported with a 95% confidence interval.

The assumptions of the Cox proportional hazards model will be investigated and tested, using Schoenfeld residuals (graphically and using the Grambsch-Therneau test – implemented using the estat phtest command in Stata) and by testing the interaction of treatment with log(survival time), i.e. including treatment as a time dependent covariate. If there is strong evidence that the proportional hazards assumption does not hold alternative methods will be used, such as using a flexible parametric survival model to estimate the restricted mean survival time at Day 90.

**Days alive and out of hospital up to 90 days (DAOH-90)**

This will be defined as follows:

- For participants who die prior to Day 90 a value of 0 will be assigned
- For participants who survive to Day 90 this will be measured as 90 – (number of days spent in hospital within 90 days of ‘trial day 1’)

‘trial day 1’ will be defined as per section 5.1.1. Participants who withdraw from trial follow-up and from providing routinely available data prior to day-90 will not be included in the analysis as their status at day-90 will be unknown.

Analyses will be performed using AS1, see section 3.2, following the ITT principle.

DAOH-90 will be summarised descriptively by randomised treatment group using the mean and standard deviation and/or median, IQR and range. The treatment effect will be estimated as the mean difference (intervention - control) by fitting a mixed-effects linear regression model, with centre included as a random effect and community-acquired versus hospital-acquired infection as a fixed effect. The treatment effect estimate will be reported with a 95% confidence interval.

The assumptions of the model will be explored, as described in section 5.1.2.

**Suspected clinically relevant antibiotic-associated adverse events**

See section 6.

**Length (in days) of critical care unit stay up to 90 days for initial index episode of sepsis**

The duration of the index critical care admission (time to discharge from critical care) will be measured as the time, in days, from the date of admission to critical care to the date of discharge from this critical care stay. Participants who die or withdraw from trial follow-up and from providing routinely available data prior to being discharged from critical care will be censored at their date of death or date of withdrawal respectively.

Analyses will be performed using AS1, see section 3.2, following the ITT principle.

Time to discharge from critical care will be summarised using Aalen-Johansen cumulative incidence functions to account for death as a competing risk. The treatment effect will be estimated using the sub-distribution HR, estimated by fitting a Fine and Gray regression model, adjusted for community-acquired versus hospital-acquired infection. The treatment effect estimate will be reported with a 95% confidence interval.

The duration of the index critical care admission will also be reported descriptively (e.g. using mean, geometric mean, median etc. as appropriate for the distribution of the data) by treatment and survival status (i.e. those who die during index critical care admission and those who are discharged alive from critical care). If mortality rates differ between treatment groups causal effect models may also be used to estimate the Survivor Average Causal Effect.

In addition, we will also measure the total time spent in critical care up to 90 days or death, including any readmissions to critical care, either during the index hospitalisation or subsequent readmissions to hospital. Differences between treatment groups will be estimated using a suitable mixed-effect model for count data, as appropriate for the distribution of the data. Recruiting centre will be included as a random effect and treatment group and other stratification factors as fixed effects. We provisionally plan to use a Negative Binomial model however we will also explore alternative models such as Poisson, or zero-inflated versions depending on which is most suitable for the distribution of the data. We will explore this graphically by plotting the observed and predicted number of events using each model, using the Pearson chi-square goodness of fit test and using Akaike's and Bayesian information criterion (AIC and BIC).

**Length (in days) of hospital stay up to 90 days**

The duration of the index hospital admission (time to discharge from hospital) will be measured as the time, in days, from the date of admission to the date of discharge from this index stay. Participants who die or withdraw from trial follow-up and from providing routinely available data prior to being discharged from hospital will be censored at their date of death or date of withdrawal respectively.

Analyses will be performed using AS1, see section 3.2, following the ITT principle.

Time to discharge from hospital will be summarised using Aalen-Johansen cumulative incidence functions to account for death as a competing risk. The treatment effect will be estimated using the sub-distribution HR, estimated by fitting a Fine and Gray regression model, adjusted for community-acquired versus hospital-acquired infection. The treatment effect estimate will be reported with a 95% confidence interval.

The duration of the index hospital admission will also be reported descriptively (e.g. using mean, geometric mean, median etc. as appropriate for the distribution of the data) by treatment and survival status (i.e. those who die during index hospital admission and those who are discharged alive from hospital). If mortality rates differ between treatment groups causal effect models may also be used to estimate the Survivor Average Causal Effect.

In addition, we will also measure the total time spent in hospital up to 90 days or death, including any readmissions to hospital. Differences between treatment groups will be estimated using a suitable mixed-effect model for count data, as appropriate for the distribution of the data. Recruiting centre will be included as a random effect and treatment group and other stratification factors as fixed effects. We provisionally plan to use a Negative Binomial model however we will also explore alternative models such as Poisson, or zero-inflated versions depending on which is most suitable for the distribution of the data. We will explore this graphically by plotting the observed and predicted number of events using each model, using the Pearson chi-square goodness of fit test and using Akaike's and Bayesian information criterion (AIC and BIC).

**Duration of initial antibiotic course for sepsis**

This will be reported as described in section 4.3.1.

**Rate of further/recurrence of infections requiring additional antibiotic courses up to 28 days.**

Further infections or recurrence of infections requiring further antibiotic treatment following the initial course for sepsis will be reported by the local site team.

The observation period will be up to 28 days from ‘trial day 1’, as defined in section 5.1.1.

Analyses will be performed using AS1, see section 3.2, following the ITT principle.

Time to first further / recurrence of infection will be measured as the time from ‘trial day 1’ to first reported further infection or recurrence of infection requiring antibiotic treatment up to Day 28. Participants who die or withdraw from trial follow-up and from providing routinely available data prior to Day 28 will be censored at their date of death or date of withdrawal respectively. Time to further / recurrence of infection will be summarised using Aalen-Johansen cumulative incidence functions to account for death as a competing risk. The treatment effect will be estimated using the sub-distribution HR, estimated by fitting a Fine and Gray regression model, adjusted for community-acquired versus hospital-acquired infection. The treatment effect estimate will be reported with a 95% confidence interval.

The number of further infections and recurrences of infections will also be reported. Descriptive data including the site of infection and pathogens detected will be summarised by randomised treatment group.

Differences between treatment groups will be estimated using a suitable mixed-effect model for count data, as appropriate for the distribution of the data. Recruiting centre will be included as a random effect and treatment group and other stratification factors as fixed effects. We provisionally plan to use a Negative Binomial model however we will also explore alternative models such as Poisson, or zero-inflated versions depending on which is most suitable for the distribution of the data. We will explore this graphically by plotting the observed and predicted number of events using each model, using the Pearson chi-square goodness of fit test and using Akaike's and Bayesian information criterion (AIC and BIC).

**Readmission to critical care or hospital during the 90 day follow up period**

The observation period will be up to 90 days from ‘trial day 1’, as defined in section 5.1.1.

Analyses will be performed using AS1, see section 3.2, following the ITT principle.

Time to first hospital readmission will be measured as the time from ‘trial day 1’ to first reported hospital readmission up to Day 90. Participants who die or withdraw from trial follow-up and from providing routinely available data prior to Day 90 will be censored at their date of death or date of withdrawal respectively. Time to hospital readmission will be summarised using Aalen-Johansen cumulative incidence functions to account for death as a competing risk. The treatment effect will be estimated using the sub-distribution HR, estimated by fitting a Fine and Gray regression model, adjusted for community-acquired versus hospital-acquired infection. The treatment effect estimate will be reported with a 95% confidence interval.

Time to critical care readmission will be reported and analysed similarly.

## Exploratory outcomes

There are no pre-specified exploratory outcome measures.

## Missing data

We do not anticipate high levels of missing data, however if more than 5% of participants, either overall or in either treatment group, withdraw from the use of routinely collected data will we will undertake sensitivity analyses to explore the robustness of results to the “missing not at random” assumption.

Patterns of missing data will be explored by summarising key baseline characteristics between those who did and did not withdraw in each treatment group.

For the co-primary outcomes we will implement controlled multiple imputation (MI) using a δ-based pattern-mixture approach, following the guide proposed by Cro *et al* [10]. Briefly, missing outcomes will be imputed under the assumption that it is MAR, conditional on the other variables in the imputation model. We initially plan to impute 50 datasets, which is very likely to be higher than the simple rule of thumb of one imputation per percent of missing data [11]. However, the number of imputations may be increased if 50 imputations is not felt to provide adequate precision. A fixed value, δ, will then be added to the imputed values to increase the mean response beyond that predicted under MAR. Each imputed dataset will be analysed using the primary analysis model and Rubin’s rules will be used to combine treatment estimates across datasets to give a single value [12]. We will also explore how extreme a value a value of δ (positive or negative) would be needed to change the interpretation of the results; this ‘tipping point’ value for δ will be reported and consideration given to how realistic or plausible this value would be in practice.

# **SAFETY**

## Adverse events

Clinically relevant antibiotic-associated adverse events (suspected antibiotic-associated AEs where antibiotic treatment has been changed or stopped due to a suspected event) will be reported from the time of randomisation to the date of discharge from hospital

Adverse events are reported by type, using the following categories:

- Anaphylaxis
- Gastrointestinal
- Haematological
- Hepatobiliary
- Renal
- Neurological
- Dermatological
- Cardiac
- Muscular
- Clostridium difficile diarrhoeal infection
- New infection with multi-drug resistant organism
- Other

‘Other’ adverse events will be coded and categorised as appropriate. Any coding will be reviewed by a medical professional.

The safety analysis set (SAS), see section 3.2, will be used for reporting adverse events.

For each adverse event, the number and proportion of participants affected will be reported. The difference between treatment groups will be estimated using the relative risk and will be reported with a 95% confidence interval.

The observation time will be calculated for each participants as the time to randomisation to discharge from hospital or death, whichever occurs first. The rate of adverse events in each treatment group will be calculated as the number of adverse events reported over the total observation time and will be reported with a 95% confidence interval.

## Serious adverse events

See section 10.2.2 of the study protocol for SAE reporting exclusions and requirements.

SAEs will be reported either in a line listing or tabulated, depending on the number reported. The number and proportion of participants reporting at least one SAE will be reported by randomisation treatment group.

## Other safety measures

Laboratory data will be summarised descriptively by randomised treatment group at each timepoint.

# **statistical software**

Statistical analyses will be carried out using Stata version 18 or later. R software may also be used, e.g. for producing graphical summaries of data.

# **references**

1. Bauer M, Gerlach H, Vogelmann T, Preissing F, Stiefel J, Adam D. Mortality in sepsis and septic shock in Europe, North America and Australia between 2009 and 2019-results from a systematic review and meta-analysis. Crit Care. 2020 May 19;24(1):239.
2. FDA. Non-Inferiority Clinical Trials to Establish Effectiveness. FDA Guid. 2016;(November).
3. Sorbello A, Komo S, Valappil T, Nambiar S. Registration Trials of Antibacterial Drugs for the Treatment of Nosocomial Pneumonia. Clin Infect Dis. 2010 Aug 1;51(S1):S36–41.
4. Spellberg B, Talbot G. Recommended design features of future clinical trials of antibacterial agents for Hospital-acquired bacterial pneumonia and ventilator-associated bacterial pneumonia. Clin Infect Dis. 2010 Aug 1;51(SUPPL. 1):150–70.
5. Chastre J, Wolff M, Fagon JY, Chevret S, Thomas F, Wermert D, et al. Comparison of 8 vs 15 days of antibiotic therapy for ventilator-associated pneumonia in adults: a randomized trial. JAMA J Am Med Assoc. 2003 Nov 19;290(19):2588–98.
6. de Jong E, van Oers JA, Beishuizen A, Vos P, Vermeijden WJ, Haas LE, et al. Efficacy and safety of procalcitonin guidance in reducing the duration of antibiotic treatment in critically ill patients: A randomised, controlled, open-label trial. Lancet Infect Dis. 2016 Jul;16(7):819–27.
7. Bouadma L, Luyt CE, Tubach F, Cracco C, Alvarez A, Schwebel C, et al. Use of procalcitonin to reduce patients’ exposure to antibiotics in intensive care units (PRORATA trial): a multicentre randomised controlled trial. Lancet. 2010 Feb 6;375(9713):463–74.
8. Pedroza C, Thanh Truong VT. Performance of models for estimating absolute risk difference in multicenter trials with binary outcome. BMC Med Res Methodol. 2016 Aug 30;16(1):113.
9. Battey, HS, Cox DR, Jackson MV. On the linear probability model for binary data. R. Soc Open Sci 6: 190067. <https://doi.org/10.1098/rsos.190067>
10. Cro S, Morris TP, Kenward MG, Carpenter JR: Sensitivity analysis for clinical trials with missing continuous outcome data using controlled multiple imputaion: A practical guide. Statistics in Medicine 2020; 39:2815-2842.
11. White IR, Royston P, Wood AM: Multiple imputation using chained equations: issues and guidance for practice. Statistics in Medicine 2011;30(4):377-399.
12. Rubin, DB: Multiple Imputation for Nonreponse in Surveys. Wiley Series in Probability and Mathematical Statistics. New York, NY: John Wiley & Sons; 1987.

# **APPENDIX**

More information is presented in **Table A1** on the infection source inducing sepsis, specifically the causative microorganisms. Data will be presented either by pathogen (as below), or by microbial classification (bacteria, fungi, and viruses) depending on the number reported.

**Table A1: Pathogens involved in the infection inducing sepsis, by randomised group.**

| Causative microorganism identified | Short course  N = | Standard of Care  N = | Overall  N = |
| --- | --- | --- | --- |
| Acinetobacter baumannii |  |  |  |
| Acinetobacter lwoffii |  |  |  |
| Aerococcus viridans |  |  |  |
| Aeromonas hydrophilia |  |  |  |
| Arcanobacterium haemolyticum |  |  |  |
| Bacillus cereus |  |  |  |
| Bacteroides fragilis |  |  |  |
| Bartonella heselae |  |  |  |
| Bartonella quintana |  |  |  |
| Bordetella pertussis |  |  |  |
| Brucella neotomae |  |  |  |
| Burkholderia cepacia |  |  |  |
| Campylobacter coli |  |  |  |
| Campylobacter jejuni |  |  |  |
| Candida (any species) |  |  |  |
| Citrobacter freundii |  |  |  |
| Clostridium difficile |  |  |  |
| Clostridium perfringens |  |  |  |
| Corynebacterium diphteriae |  |  |  |
| Corynebacterium jeikeium |  |  |  |
| Corynebacterium urealyticum |  |  |  |
| Eikenella corrodens |  |  |  |
| Enerococcus faecalis |  |  |  |
| Enerococcus faecium |  |  |  |
| Enterobacter aerogenes |  |  |  |
| Enterobacter cloacae |  |  |  |
| Escherichia coli |  |  |  |
| Fusobacterium nucleatum |  |  |  |
| Haemophilus influenzae |  |  |  |
| Helicobacter pylori |  |  |  |
| Influenza virus A |  |  |  |
| Klebsiella oxytoca |  |  |  |
| Klebsiella pneumoniae |  |  |  |
| Lactobacillus acidophilus |  |  |  |
| Legionella pneumophilia |  |  |  |
| Listeria monocytogenes |  |  |  |
| Microbacterium sp. |  |  |  |
| Micrococcus luteus |  |  |  |
| Moraxella catarrhalis |  |  |  |
| Morganella morganii |  |  |  |
| Mycobacterium chelonae |  |  |  |
| Mycoplasma bovis |  |  |  |
| Neisseria meningitidis |  |  |  |
| Nocardia nova |  |  |  |
| Pasteurella multocida |  |  |  |
| Propionibacterium acnes |  |  |  |
| Proteus mirabilis |  |  |  |
| Proteus vulgaris |  |  |  |
| Providencia stuartii |  |  |  |
| Pseudomonas aeruginosa |  |  |  |
| Rothia dentocariosa |  |  |  |
| Salmonella enterica |  |  |  |
| SARS-CoV-2 |  |  |  |
| Serratia marcescens |  |  |  |
| Shigella sonnei |  |  |  |
| Staphylococcus aureus (MRSA) |  |  |  |
| Staphylococcus aureus (MSSA) |  |  |  |
| Staphylococcus epidermidis |  |  |  |
| Staphylococcus haemolyticus |  |  |  |
| Staphylococcus lugdunensis |  |  |  |
| Staphylococcus saprophyticus |  |  |  |
| Stenotrophomonas maltophilia |  |  |  |
| Streptococcus agalactiae |  |  |  |
| Streptococcus gordonii |  |  |  |
| Streptococcus mutans |  |  |  |
| Streptococcus pneumoniae |  |  |  |
| Streptococcus pyogenes |  |  |  |
| Veillonella dispar |  |  |  |
| Vibrio parahaemolyticus |  |  |  |
| Yersinia pseudotuberculosis |  |  |  |
