# Supplementary material for: SHORTER trial: protocol for a pragmatic, multicentre, randomised controlled trial of short-duration antibiotic therapy for critically ill patients with sepsis
Source: BMJ Open. 2026 Mar 26;16(3):e117142. doi: 10.1136/bmjopen-2026-117142 (PMC13034387; doi:10.1136/bmjopen-2026-117142)
Supplement: Supplementary file 8 [file bmjopen-2026-117142.reviewer_comments.pdf]

PEER REVIEW HISTORY

BMJ Open publishes all reviews undertaken for accepted manuscripts. Reviewers are asked to complete a checklist review form and are provided with free text boxes to elaborate on their assessment. These free text comments are reproduced below.

ARTICLE DETAILS

Title (Provisional)

SHORTER Trial: protocol for a pragmatic, multicentre, randomised controlled trial of short duration antibiotic therapy for critically ill patients with sepsis.

Authors

Mawson, Phil; Morton, Miranda; Walmsley, Zoe; Wafer, Rebecca; Hancock, Helen C; Mossop, Helen; Al-Ashmori, Sarah; Emerson, Lydia M; Smith, Jamie; Colquhoun (formerly Wojcik), Dr Gosha; Wu, Hangjian; Fernandez-Garcia, Cristina; Ternent, Laura; Kumar, Rashmi; Ogden , Margaret; Loraine, Laura; Taylor, Leia; Ndlovu, Sipho; Hall, Phil; Gordon, Anthony C; Rostron, Anthony; McMullan, Ronan; Felton, Tim; Walsh, Timothy; McAuley, Daniel Francis; Dark, Paul; Simpson, A John; Hellyer, Thomas P

This article was not externally reviewed at BMJ Open. Protocols that have been independently assessed prior to submission to BMJ Open are usually fast-tracked to publication on the grounds that further substantial changes will not be possible. This independent assessment will usually be external, independent review for both a substantial grant award from a non-commercial or government funder and ethics approval.
